# Characterization of the Metabolic Pathways of 4-Chlorobiphenyl (PCB3) in HepG2 Cells Using the Metabolite Profiles of Its Hydroxylated Metabolites

**DOI:** 10.1021/acs.est.1c01076

**Published:** 2021-06-14

**Authors:** Chun-Yun Zhang, Susanne Flor, Patricia Ruiz, Gabriele Ludewig, Hans-Joachim Lehmler

**Affiliations:** † Department of Occupational and Environmental Health, The University of Iowa, Iowa City, Iowa 52242, United States; ‡ Office of Innovation and Analytics, Simulation Science Section, Agency for Toxic Substances and Disease Registry, Atlanta, Georgia 30333, United States

**Keywords:** HepG2 cells, hydroxylated metabolites, 4-chlorobiphenyl

## Abstract

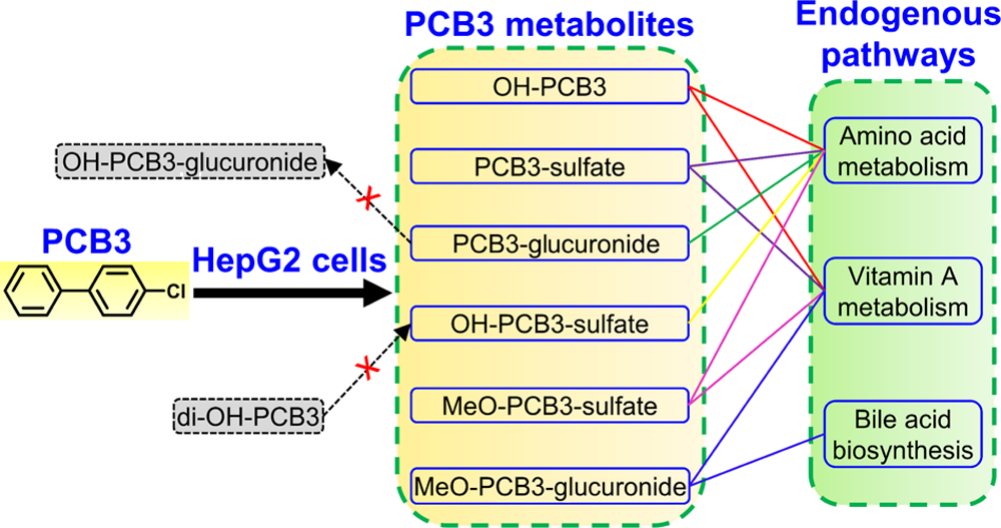

The characterization of the metabolism of lower chlorinated PCB, such as 4-chlorobiphenyl (PCB3), is challenging because of the complex metabolite mixtures formed *in vitro* and *in vivo*. We performed parallel metabolism studies with PCB3 and its hydroxylated metabolites to characterize the metabolism of PCB3 in HepG2 cells using nontarget high-resolution mass spectrometry (Nt-HRMS). Briefly, HepG2 cells were exposed for 24 h to 10 μM PCB3 or its seven hydroxylated metabolites in DMSO or DMSO alone. Six classes of metabolites were identified with Nt-HRMS in the culture medium exposed to PCB3, including monosubstituted metabolites at the 3′-, 4′-, 3-, and 4- (1,2-shift product) positions and disubstituted metabolites at the 3′,4′-position. 3′,4′-Di-OH-3 (4′-chloro-3,4-dihydroxybiphenyl), which can be oxidized to a reactive and toxic PCB3 quinone, was a central metabolite that was rapidly methylated. The resulting hydroxylated-methoxylated metabolites underwent further sulfation and, to a lesser extent, glucuronidation. Metabolomic analyses revealed an altered tryptophan metabolism in HepG2 cells following PCB3 exposure. Some PCB3 metabolites were associated with alterations of endogenous metabolic pathways, including amino acid metabolism, vitamin A (retinol) metabolism, and bile acid biosynthesis. In-depth studies are needed to investigate the toxicities of PCB3 metabolites, especially the 3′,4′-di-OH-3 derivatives identified in this study.

## Abstract 2

Please provide a synopsis

## Introduction

Polychlorinated biphenyls (PCBs) are a class of human-made chemicals containing a biphenyl moiety substituted with 1 to 10 chlorine atoms. PCBs were used in dielectric fluids, coolants, lubricants, paints, plastics, adhesives, and sealants. Their production was banned in the United States in the late 1970s,^1^ but PCBs can still be used in totally enclosed applications. Notably, some PCBs are still inadvertently produced and can be found in paints and other consumer products.^2−4^ PCBs persist in the environment and are transported over long distances because of their semivolatile nature, and they bioaccumulate and biomagnify in terrestrial food chains. As a result, PCBs are detected in most environmental media, such as indoor and outdoor air,^5^ soil,^6^ water,^7^ sediments,^8^ human food,^9^ and ultimately the human body.^10^ Exposure to PCBs is associated with adverse health effects, for example, immunotoxicity,^11^ cancer,^12−14^ and neurodevelopmental disorders.^15,16^

PCB3, a lower chlorinated PCB congener, is a component of several commercial PCB mixtures.^17^ It is detected in the environment^18,19^ and human blood.^20^ Unlike higher chlorinated PCB congeners, PCB3 is readily metabolized to hydroxylated PCB3 (OH-PCB3) metabolites and PCB3 sulfates in rats exposed to PCB3.^21−23^*In vitro* studies with rat liver microsomes demonstrate that PCB3 undergoes biotransformation at the *meta* and *para* positions of the nonchlorinated phenyl ring.^24,25^ Redox reactive dihydroxylated PCB3 metabolites, such as PCB3 hydroquinones or catechols, were also identified in metabolism studies with rat liver microsomes^25^ and disposition studies in rats.^21−23^ These OH-PCB3 metabolites are further metabolized to PCB3 sulfates and glucuronides *in vitro*.^26,27^

Like other PCB metabolites, the metabolic activation of PCB3 to diverse oxidative metabolites and the corresponding conjugates is implicated in PCB3-mediated toxicities.^28^ For example, hydroxylated PCB3 metabolites and the corresponding sulfate conjugates are high-affinity ligands of transthyretin,^27,29,30^ indicating that these metabolites are endocrine-disrupting chemicals interfering with thyroxine and vitamin A transport. Metabolic activation of PCB3 can result in reactive metabolites, such as arene oxides or redox-active quinone metabolites. These metabolites form adducts with DNA and proteins *in vitro*(31−33) and *in vivo*.^34^ PCB3 metabolites cause DNA strand breaks,^35^ chromosome loss,^36^ polyploidization,^37^ sister chromatid exchange (SCE) induction,^37^ and gene mutations^36^ in cells in culture. Gene mutations and initiating activity of PCB3, likely involving the metabolic activation of PCB3, have been observed *in vivo*.^38−40^

The use of rat models represents a limitation of studies of the metabolism and toxicity of PCB3 due to species differences in the oxidative metabolism, as has been shown for several higher chlorinated PCBs.^41,42^ Despite the use of human cell lines for PCB3 toxicity studies,^43,44^ the metabolism of PCB3 has not been characterized in human models. It is unknown to which degree the PCB3 metabolites formed in humans differ from those formed in rats. Basic characterization of the human-relevant metabolism of PCB3 and other congeners is a first step in characterizing their toxicokinetics and understanding body burdens. This information, in turn, is needed to manage the risks associated with current exposures to PCBs.^45^

Here, we characterize the metabolite profiles of PCB3 and its hydroxylated metabolites formed by the HepG2 cells using Nt-HRMS. Untargeted metabolomic analyses were employed to identify the changes in endogenous metabolites and metabolic pathways following PCB3 exposure and revealed associations between specific PCB3 metabolites and endogenous metabolic pathways.

## Experimental Section

### *In Silico* Metabolite Predictions with ADMET Predictor and MetaDrug

The *in silico* metabolite predictions were performed with ADMET Predictor (Simulations Plus, Lancaster, CA, USA) and MetaDrug (Clarivate Analytics, New York, NY, USA) as described in the Supporting Information.^46^

### Exposure of HepG2 Cells to PCB3 or Its Metabolites

HepG2 cells (6 × 10^6^/well) in 3 mL of complete minimum essential medium (MEM) were seeded into 6-well plates. For additional information regarding cell culture supplies, the HepG2 cells and their maintenance, and the sources and authentication of test compounds, see the Supporting Information. After 48 h of attachment, cells were exposed in parallel to PCB3, 2′-OH-3, 3′-OH-3, 4′-OH-3, 2-OH-3, 3-OH-3, 4-OH-2, or 3′,4′-di-OH-3 in an exposure medium. These OH-PCB3 derivatives include all six possible monohydroxylated PCB3 metabolites. Exposure experiments were performed without fetal bovine serum (FBS) to facilitate the partitioning of PCB3 and its metabolites into the cells. Instead, cells were cultured with 4.5 mM D-glucose (3 mL per well, 0.1% DMSO). All experiments were performed in triplicate. Based on similar metabolism and toxicity studies,^46,47^ a concentration of 10 μM was used for all test compounds to facilitate the detection of minor PCB3 metabolites. HepG2 control cells were exposed to the exposure medium containing 0.1% DMSO. After incubation, the media were transferred into glass vials, and the cells were washed once with PBS (1 mL). The cells were harvested into PBS (1 mL) with a rubber policeman and collected into a separate glass vial. The wells were washed once with PBS (1 mL). The vials with medium and cells, combined with the respective PBS wash, were stored at −20 °C until analysis. The results from parallel cytotoxicity studies are presented in the Supporting Information (Figure S1).

### Extraction of PCB3 and Its Metabolites from the Medium

The exposure media (∼4 mL) were spiked with PCB14 (1000 ng), 3-F-4′-PCB3 sulfate (100 ng), and 3-F-4′-OH-PCB3 (100 ng) as surrogate recovery standards, as described,^23^ and acidified with 10% formic acid (400 μL). Acetonitrile (3 mL) was added to the samples, followed by magnesium sulfate (1.2 g) and sodium chloride (0.3 g). The vials were inverted for 5 min and centrifuged at 1811*g* for 5 min. The acetonitrile layers were transferred to spin filters. The aqueous phases were re-extracted with acetonitrile (1 mL), and the organic layers were added to the spin filters. The spin filters were inverted for 5 min and centrifuged at 1811*g* for 5 min. For experiments with PCB3, aliquots (100 μL) of the eluents were spiked with PCB15 (100 ng) as an internal standard for PCB3 analysis (see the Supporting Information). The remaining extracts were evaporated to dryness under a gentle stream of nitrogen and reconstituted in acetonitrile/water (200 μL; 15:85, vol/vol) for PCB3 metabolite analysis using liquid chromatography–mass spectrometry (LC–MS). The whole extracts were utilized for PCB3 metabolite analyses for medium samples from incubations with OH-PCB3 metabolites.

Selected cell pellet samples were extracted analogously. Similar to earlier studies with HepG2 cells,^46,48^ the levels of the metabolites were lower in these extracts than extracts from the media, with several metabolites being below the detection limit of our analytical method. Therefore, cell pellets were not analyzed further.

### LC–MS Analysis

An initial screening for PCB3 metabolites was performed on an ultraperformance liquid chromatograph (UPLC) (Waters Acquity UPLC, Milford, MA, USA) coupled with a quadrupole time-of-flight mass spectrometer (LC-QT of MS; Waters Q-Tof Premier, Milford, MA, USA) at the High-Resolution Mass Spectrometry Facility of the University of Iowa (Iowa City, IA, USA). Medium samples were subsequently analyzed with a UPLC (Ultimate 3000 UHPLC+ Focused, Thermo Fisher, Waltham, MA, USA) coupled with a Q exactive hybrid quadrupole-Orbitrap mass spectrometer (LC-Orbitrap MS; Thermo Fisher) at the Center of Mass Spectrometry and Proteomics at the University of Minnesota (Minneapolis, MN, USA) using full scan and MS/MS methods. For more details about the LC–MS analysis, see the Supporting Information. Full scan raw data were converted to an mzXML format with Proteowizard software. PCB3 metabolites were identified with XCMS Online and further confirmed based on their accurate mass and isotopic mass patterns with Thermo Xcalibur software and their MS/MS data, as described.^46,49^ For a summary of these data, see Table S1. For quality assurance/quality control information, see the Supporting Information.

### Metabolomic Analysis

Metabolomic analysis was performed following an earlier report and as described in the Supporting Information.^46^ For a metabolome-wide association analysis with PCB3 metabolite classes, the sum of the peak areas of all isomers of each PCB3 metabolite class was determined from the extracted ion chromatograms of all samples, including controls, with a mass window of 10 ppm. The peak areas of the PCB3 metabolites were normalized by the total intensities and log_2_ transformed before they were used for association analyses using a linear model. Pathway enrichment analyses were performed in *mummichog*(50) with an input feature list considering *p* < 0.05 as significant features. If applicable, analyses were performed in R (version 3.6.3).

## Results and Discussion

### Formation of PCB3 Metabolites in the Cell Culture Medium of HepG2 Cells Exposed to PCB3

We used HepG2 cells, a human hepatoma cell line, as an inexpensive model system to identify PCB3 metabolites potentially formed in humans. The HepG2 cell line readily metabolizes lower chlorinated PCBs, such as PCB11, but not higher-chlorinated PCBs,^49^ to complex mixtures of oxidative metabolites and their conjugates^46^ because of the lower expression of xenobiotic processing genes, including the cytochrome P450 isoforms involved in PCB metabolism,^41,51−53^ compared to human primary hepatocytes.^54^ The slower PCB metabolism in HepG2 cells also provides a better time window to investigate the complex PCB metabolite mixtures formed in hepatocytes.

The PCB metabolites in the culture medium from HepG2 cells exposed to 10 μM PCB3 were characterized with Nt-HRMS. A screening list developed with *in silico* predictions was used to guide the metabolite identification. For more information about the *in silico* prediction results of the metabolism of PCB3 and selected metabolites, see the Supporting Information (Tables S2 and S3). We identified three classes of PCB3 metabolites, including monohydroxylated PCB3 metabolites and their sulfate and glucuronide conjugates, sulfates of dihydroxylated PCB3, and sulfates and glucuronides of methoxylated-hydroxylated PCB3 in the culture medium (Tables S1 and S4). Consistent with its rapid metabolism, PCB3 levels in cell culture media decreased with incubation time (Figure S2).

#### OH-PCB3 Metabolites and Their Conjugates

**Figure 1:**
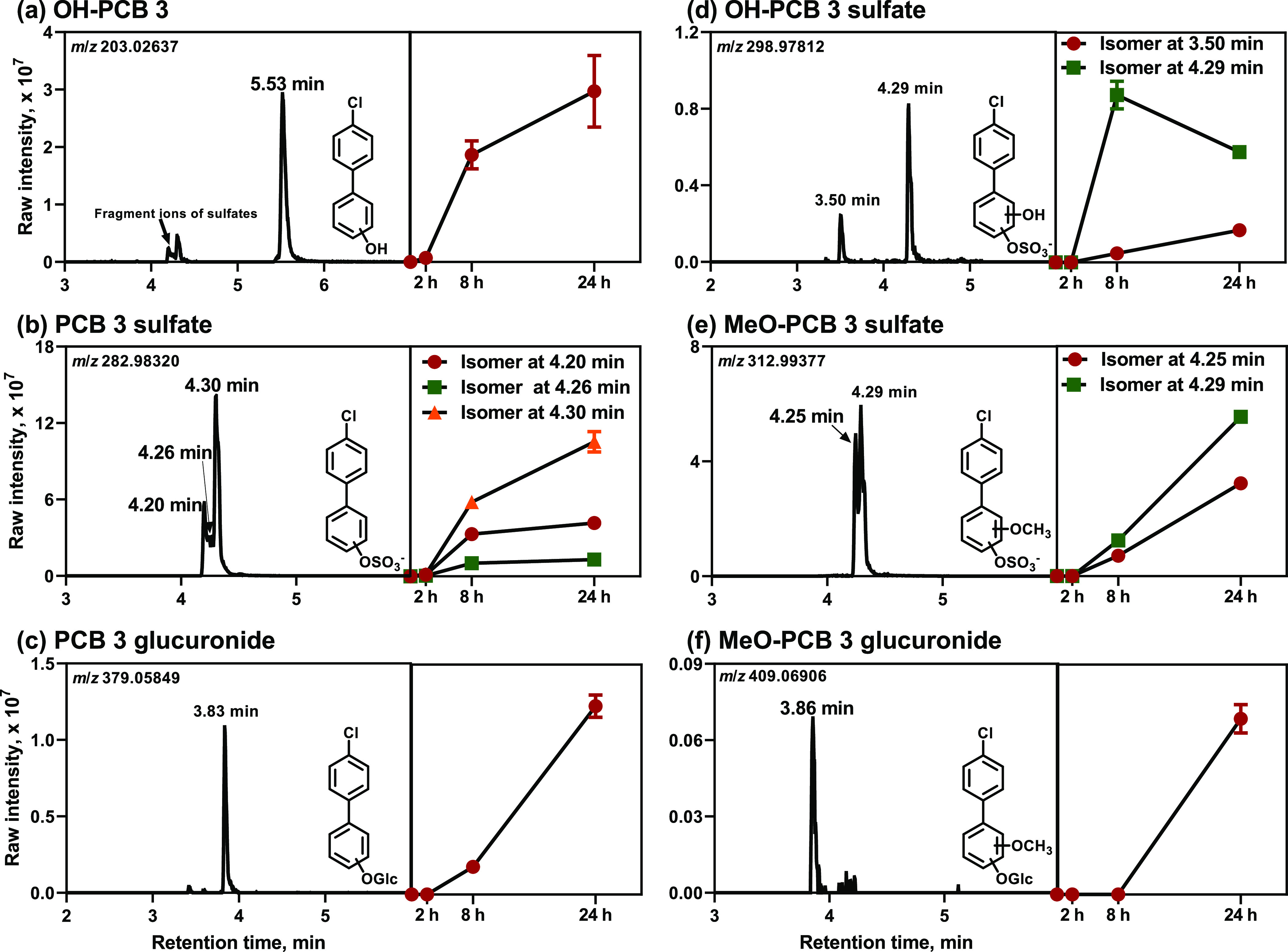
Six classes of metabolites were formed in HepG2 cells exposed to PCB3, including (a) OH-PCB3 (*m*/*z* 203.02637), (b) PCB3 sulfate (*m*/*z* 282.98320), (c) PCB3 glucuronide (*m*/*z* 379.05849), (d) OH-PCB3 sulfate (*m*/*z* 298.97812), (e) MeO-PCB3 sulfate (*m*/*z* 312.99377), and (f) MeO-PCB3 glucuronide (*m*/*z* 409.06906) metabolites. LC-Orbitrap MS analyses were performed in the negative mode. The extracted ion chromatograms are based on the calculated accurate mass of each metabolite class, with a mass window of 10 ppm. The levels of each metabolite class were semi-quantitatively calculated as the metabolite peak area/internal standard peak area × 100. The placement of the functional groups on different phenyl rings is for illustration purposes only and does not indicate their actual positions. For selected MS and MS/MS spectra, see the Supporting Information (Figures S3–S8). Glc, glucuronide.

We observed one OH-PCB3 metabolite (Figure 1a and Figure S3), three peaks of PCB3 sulfate metabolites (Figure 1b and Figure S4), and one peak of PCB3 glucuronide metabolite (Figure 1c and Figure S5) in the culture medium from HepG2 cells. The levels of these metabolites typically increased over time (Figure 1a,c). The levels of OH-PCB3 and their sulfate conjugates, but not glucuronide conjugates, plateaued beginning with the 8 h time point, possibly due to their biotransformation to other metabolites, for example, hydroxylated PCB3 sulfates, as observed in rats.^55^

The presence of the three peaks of PCB3 sulfates suggests that at least three OH-PCB3 isomers were formed by HepG2 cells; however, only one OH-PCB3 metabolite peak was observed. Likely, other OH-PCB3 isomers were not detected because of their rapid biotransformation or co-elution issues, as reported previously.^21^ The formation of OH-PCB3 by HepG2 cells confirms the formation of several metabolites predicted by MetaDrug and, to a lesser extent, ADMET Predictor (Tables S2 and S3). OH-PCB3 metabolites are also commonly detected metabolites of lower chlorinated PCBs in *in vitro*(25,46) and *in vivo* experiments^22,23,55,56^ and human biomonitoring studies.^57^

#### Dihydroxylated PCB3 Metabolites (di-OH-PCB3) and Their Conjugates

We observed two peaks corresponding to hydroxylated PCB3 sulfates in the cell culture media from HepG2 cells exposed for 8 and 24 h to PCB3 (Figure 1d and Figure S6). These metabolites can be formed by sulfation of a di-OH-PCB3 metabolite or oxidation of PCB3 sulfate metabolites, as observed in rats.^55^ The levels of the minor hydroxylated PCB3 sulfate metabolite increased with the increasing incubation time (Figure 1d), whereas the levels of the major metabolite initially increased and then decreased after 8 h. This metabolite may undergo further oxidative metabolism, as observed for PCB11,^46^ or be deconjugated by HepG2 cells. Similarly, deconjugation products were observed in rats exposed to PCB11 sulfate.^55^ Moreover, PCB sulfates can be deconjugated in cells in culture.^48^ We did not observe the formation of glucuronide conjugates of di-OH-PCB3 in experiments with PCB3.

The detection of catechol-derived methoxylated metabolites (see the text below) indicates that HepG2 cells formed PCB3 catechol metabolites, such as 3′,4′-di-OH-3. We did not detect PCB3 catechols in the cell culture medium, possibly because of their rapid biotransformation to sulfated or methoxylated metabolites or highly reactive PCB quinone metabolites.^31,35,40,58−61^ In contrast, three dihydroxylated PCB3 isomers were formed by rat liver microsomes, possibly due to the lack of phase 2 metabolism in the rat microsomal metabolism studies.^31^ 3′,4′-Di-OH-3, a catechol metabolite, was also detected in the bile and urine of rats exposed to PCB3 through inhalation.^21^

#### Methoxylated-Hydroxylated PCB3 Metabolites (MeO-OH-PCB3) and Their Conjugates

We detected two methoxylated PCB3 sulfates (Figure 1e and Figure S7) and one methoxylated PCB3 glucuronide (Figure 1f and Figure S8) in the media from HepG2 cell exposed to PCB3. The methoxylated metabolites formed with a delay compared to other metabolites (Figure 1). For example, the methoxylated PCB3 glucuronide was only detected at the 24 h time point. We observed both methoxylated sulfate and glucuronide metabolites in our earlier metabolism study with PCB11 in HepG2 cells.^46^ We did not detect the corresponding MeO-OH-PCB3 metabolites (i.e., methylation products of the catechol metabolite^62,63^), probably because these compounds were rapidly biotransformed to sulfate and glucuronide conjugates. MeO-OH-PCB3 metabolites have been observed in the excreta from rats,^64^ rabbits,^65^ and guinea pigs^66^ but have not been detected in humans. Methoxylated and hydroxylated PCB metabolites and their sulfate conjugates were also reported in mice exposed to a PCB mixture.^67^ Overall, HepG2 cells formed several metabolite classes that were also observed in rodent models. Further work is needed to confirm the presence of these metabolites in human biomonitoring studies.

We observed fewer PCB3 metabolites compared to our study with PCB11.^46^ We detected three subclasses of disubstituted metabolites (metabolites derived from di-OH-PCB3 or MeO-OH-PCB3) in the medium from PCB3-exposed HepG2 cells. In contrast, we observed six subclasses of disubstituted metabolites in experiments with PCB11 exposed HepG2 cells.^46^ Moreover, we did not detect any metabolites derived from trihydroxylated PCB3 isomers. In contrast, trihydroxylated metabolites were formed under identical experimental conditions from PCB11.^46^ This observation suggests that HepG2 cells metabolize lower chlorinated PCBs in a congener-specific manner. Congener-specific differences in the metabolism of lower chlorinated PCBs have been documented for studies with purified rat cytochrome P450 enzymes, rat liver microsomes, or liver tissue slices from mice.^24,68−70^ In-depth metabolism studies with lower chlorinated PCBs have not been reported for human model systems; however, higher chlorinated PCBs are metabolized in a congener-specific manner by human cytochrome P450 enzymes.^41,42,71,72^

It is noteworthy that, based on the number of metabolite classes observed, the monochlorinated PCB3 is less readily metabolized by HepG2 cells than the dichlorinated PCB11. Typically, lower chlorinated PCBs are more rapidly metabolized than higher chlorinated PCBs.^73^ However, PCB congeners with *para* chlorine substituents are more resistant to metabolism. This *para* chlorine group possibly reduces the rate of (oxidative) metabolism of both PCB3 and its metabolites compared to *meta* chlorinated PCBs, such as PCB11.^46^ It is currently unknown how *para* chlorine substituents affect the further metabolism of PCB metabolites.

### Probing the PCB3 Metabolism Pathway with the Metabolite Profiles of PCB3 Metabolites

The structure of PCB3 metabolite isomers formed by HepG2 cells cannot be identified based on the Nt-HRMS analysis (Table S1) alone because authentic standards are not available. We performed parallel metabolism studies with a set of well-authenticated hydroxylated PCB3 metabolites, including 2′-OH-3, 3′-OH-3, 4′-OH-3, 2-OH-3, 3-OH-3, 4-OH-2, and 3′,4′-di-OH-3, to overcome this limitation by comparing the metabolite profiles. We performed these analyses by LC-QTof MS because of the better chromatographic separation of the PCB3 metabolite isomers on this system.

**Figure 2:**
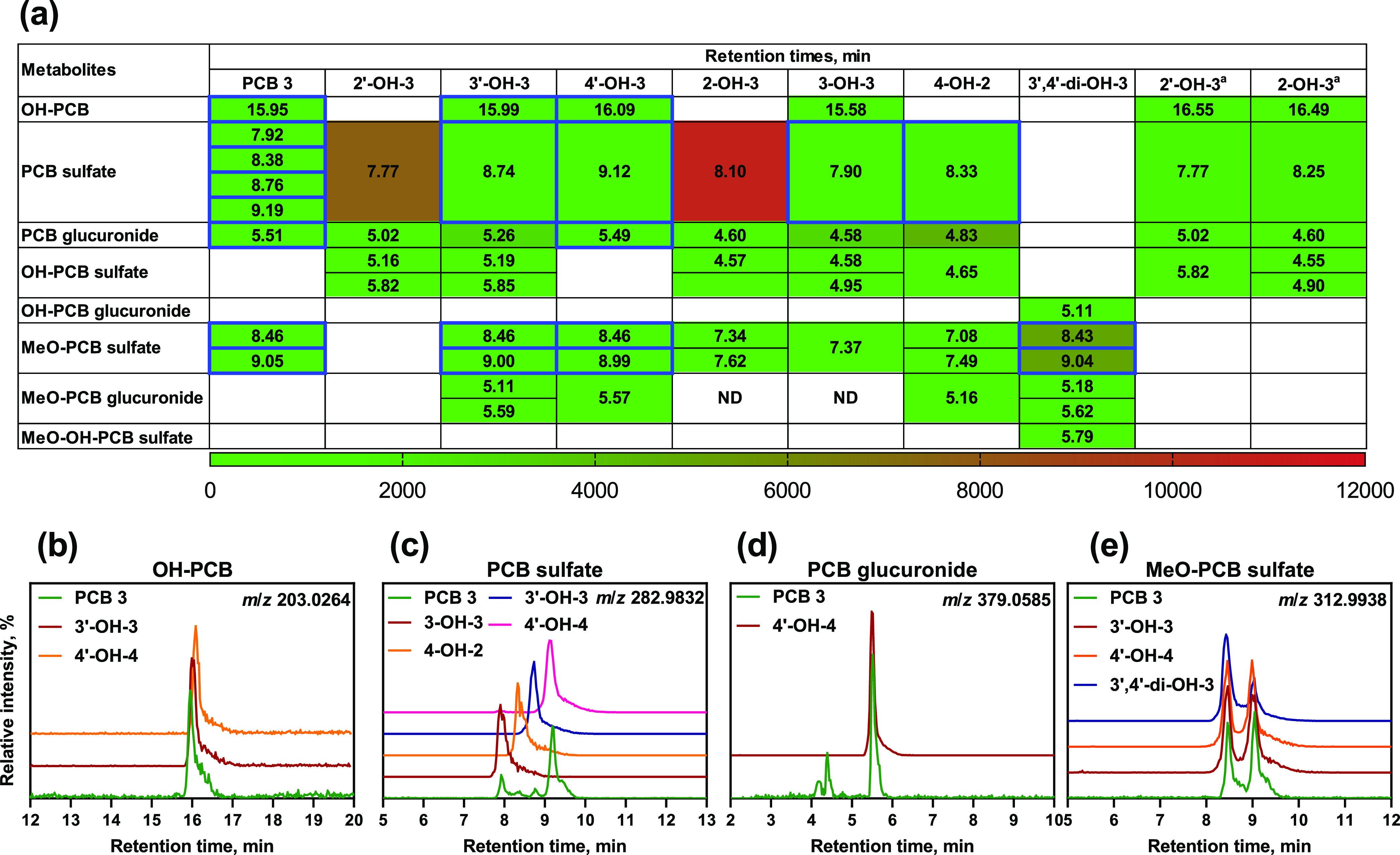
(a) Comparison of the retention times (numbers) and normalized intensities (coded colors) of the metabolites formed from PCB3 and seven hydroxylated PCB3 metabolites allowed the identification of four classes of PCB3 metabolites formed by HepG2 cells. Boxes with blue borders indicate that metabolites are also formed by HepG2 cells exposed to PCB3. The normalized intensities are shown in panel (a) as the intensity of the metabolite peak area/internal standard peak area × 100. A white box indicates that a metabolite class was not detected. Based on this comparison, the human-relevant PCB3 metabolites were identified as follows: (b) OH-PCB3 as 3′-OH-3 or 4′-OH-3, (c) sulfate as the sulfates of 3-OH-3, 4-OH-2, 3′-OH-3, and 4′-OH-3, (d) PCB3 glucuronide as 4′-OH-3, and (e) MeO-PCB3 sulfate as 4′-chloro-3-methoxy-4-sulfooxy-biphenyl and 4′-chloro-4-methoxy-3-sulfooxy-biphenyl. The formations of 3′-OH-3, 4′-OH-3, 3′-PCB3 sulfate, and 4′-PCB3 sulfate were confirmed with authentic standards. HepG2 cells were incubated in triplicate for 2 or 24 h with PCB3, 2′-OH-3, 3′-OH-3, 4′-OH-3, 2-OH-3, 3-OH-3, 4-OH-2, or 3′,4′-di-OH-3 individually (^a^ indicates incubations with 2 h time points). Metabolites were extracted from the media and analyzed as described in the Experimental Section. Analyses were performed in the negative mode on an LC-QTof MS; for additional details, see the Experimental Section. For the MS spectra of the four classes of PCB3 metabolites identified on LC-QTof MS, see the Supporting Information (Figures S9–S12).

Comparison of the metabolite profiles in the medium from incubations with the OH-PCB3 metabolites and PCB3 demonstrates that the OH-PCB3 metabolites and the corresponding conjugates have the functional groups on the 3-, 3′-, or 4′-position of PCB3 or the 4-position of PCB 2 (1,2-shift product) (Figure 2). The *ortho* hydroxylated PCB isomers (i.e., 2- and 2′-OH-3) and the corresponding conjugates were not observed in experiments with PCB3 (Figure 2a). Consistent with faster oxidation of the nonchlorinated phenyl ring, 3′- and/or 4′-OH-3 were the major monohydroxylated PCB3 metabolites formed from PCB3. The PCB3 glucuronide metabolite was identified as 4′-PCB3 glucuronide (Figure 2d). 2′-OH-3 and 2-OH-3 were more readily metabolized to the corresponding sulfate metabolites than the *meta* and *para* hydroxylated isomers. In contrast, 3′-OH-3, 3-OH-3, and 4-OH-2 were preferentially metabolized to glucuronide metabolites. 4′-OH-3 was not as readily conjugated as the other OH-PCB3 isomers (Figure 2a). These results were consistent with enzyme kinetic studies, which report that 2′-OH-3 and 3′-OH-3 have higher specificity toward human SULT1A1 than 4′-OH-3^27^ and that 2′-OH-3 has the highest specificity toward rat hepatic microsomal UGTs, followed by 3′-OH-3 and 4′-OH-3.^26^ Our results suggest that *ortho*-hydroxylated PCB3 metabolites are preferentially metabolized to sulfate metabolites, whereas *meta*-hydroxylated PCB3 metabolites more likely form glucuronide metabolites.

Glucuronide conjugates were observed for all OH-PCB3 isomers. However, no OH-PCB3 glucuronide metabolite was detected in experiments with OH-PCB3 metabolites (Figure 2a). This observation is consistent with the predictions with ADMET Predictor (Table S2) and MetaDrug (Table S3). Thus, OH-PCB3 glucuronides are likely formed by the glucuronidation of dihydroxylated PCB metabolites and not by the oxidation of PCB glucuronides. Experiments with 3′,4′-di-OH-3 confirmed that OH-PCB3 glucuronides are formed by the glucuronidation of dihydroxylated PCB metabolites in HepG2 cells (Figure 2a). No sulfated metabolites were observed in incubations with 3′,4′-di-OH-3 (Figure 2a), consistent with MetaDrug predictions (Table S3). Thus, hydroxylated PCB3 sulfate metabolites were formed by oxidation of the corresponding PCB sulfate. We cannot exclude the possibility that the sulfation of PCB3 hydroquinones forms hydroxylated PCB3 sulfates. These PCB3 hydroquinone metabolites have been reported in a metabolism study with rat liver microsomes.^25^

Experiments with 3′,4′-di-OH-3 revealed that this metabolite was metabolized to two MeO-PCB3 sulfates and, to a lesser extent, a methoxylated PCB3 glucuronide (Figure 2a). The MeO-PCB3 sulfates derived from PCB3 catechol metabolites observed in incubations with PCB3 were identified as derivatives of 3′,4′-di-OH-3 (Figure 2e). This catechol metabolite and its conjugates are also major metabolites formed in rats.^21,22^ Analogously, the MeO-PCB3 glucuronide metabolites likely correspond to 3′-MeO-4′-PCB3 or 4′-MeO-3′-PCB3 glucuronide. Unlike the parent PCB3, 3′,4′-di-OH-3 was further oxidized, resulting in a methoxylated-hydroxylated PCB3 sulfate (Figure 2a). We also observed trihydroxylated PCB11 conjugates in analogous experiments in HepG2 cells.^46^

The observation that PCB3 is oxidized in the *meta* or *para* position is consistent with studies demonstrating that higher chlorinated PCBs are preferentially oxidized in *meta* and *para* positions in studies with recombinant human cytochrome P450 enzymes or human liver microsomes.^41,42,71,72,74,75^ Similarly, the oxidation of PCB metabolites at the *para* or *meta* position is commonly observed in mammalian model systems, both *in vitro* and *in vivo*.^21−24,41,46,56,76^ Studies with rats exposed to PCB3 through inhalation identified 3′-, 4′-, and 3-PCB3 sulfate isomers and 3′,4′-di-OH-3 conjugates.^21,22^ Interestingly, rats receiving an intraperitoneal injection of PCB3 excreted 2′-, 3′-, and 4′-PCB3 sulfate in the urine, suggesting that *ortho*-hydroxylated PCB3 metabolites are formed in rats *in vivo*.^23^ 2′-OH-3, 3′-OH-3, and 4′-OH-3, together with two unidentified monohydroxylated metabolites, were observed in a metabolism study with rat liver microsomes.^25^ At least some monohydroxylated PCB3 metabolites are formed via an arene oxide intermediate, followed by a 1,2-shift, as indicated by the formation of 4-PCB 2 sulfate. Similarly, 1,2-shift metabolites are formed from other PCB congeners by human cytochrome P450 enzymes.^41,42,71,72,77^ Overall, our results confirm that HepG2 cells metabolize lower chlorinated PCBs, such as PCB3, in a manner that shows some similarities to rats.

### Metabolic Pathway of PCB3 and Its Toxicological Implications

**Figure 3:**
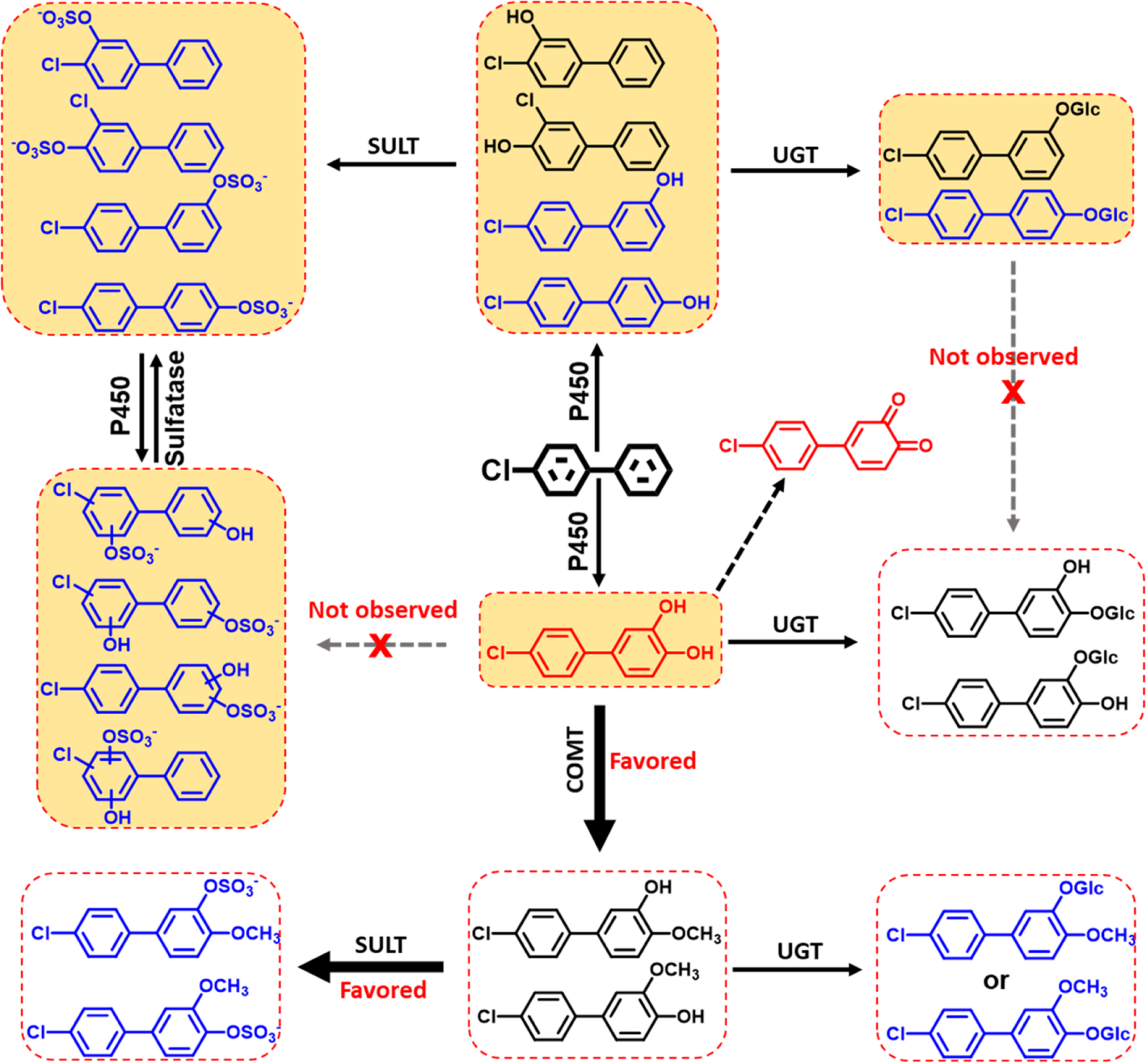
Pathways proposed for the metabolism of PCB3 in HepG2 cells. Structures shown in blue were detected in this study. Structures shown in black and red were not detected, but their formation is expected based on the available evidence from this and other studies.^46^ Catechol metabolites and their quinone derivatives are shown in red. These metabolites are likely reactive and highly toxic. Metadrug predicted metabolites with orange backgrounds. P450: cytochrome P450 enzyme; Glc: glucuronide; SULT: sulfotransferase; UGT: uridine 5′-diphospho-glucuronosyltransferase; COMT: catechol-*O*-methyltransferase.

We propose the metabolism pathway shown in Figure 3 for the PCB3 metabolism in HepG2 cells based on our experimental findings. Briefly, PCB3 is oxidized to *meta-* or *para*-OH-PCB3. Further oxidation results in the formation of PCB3 catechol metabolites, such as 3′,4′-di-OH-3. Subsequently, OH-PCB3 metabolites are biotransformed by SULTs and UGTs to sulfate and glucuronide conjugates. PCB3 sulfates but not PCB3 glucuronides can be further oxidized to hydroxylated compounds and the corresponding downstream metabolites.

3′,4′-Di-OH-3 appears to be a pivotal PCB3 metabolite that is only transiently formed in HepG2 cells. This metabolite is methylated to methoxylated-hydroxylated PCB3 metabolites, followed by conjugation to form MeO-PCB3 sulfate and MeO-PCB3 glucuronide conjugates. 3′,4′-Di-OH-3 can also be converted to OH-PCB3 glucuronides. It is unclear to which extent these metabolic pathways prevent the oxidation of 3′,4′-di-OH-3 to the corresponding PCB3 quinone. Studies in the resistant hepatocyte model demonstrated that this quinone acts as the ultimate carcinogenic metabolite resulting from the bioactivation of PCB3 in rat liver.^40^ It is also unknown to which extent PCB3 quinone adducts were formed with cellular nitrogen and sulfur nucleophiles, including proteins and DNAs,^31,32,34^ in HepG2 cells. Future studies are needed to confirm the proposed metabolic pathway of PCB3 and characterize the potential toxicities associated with the formation of 3′,4′-di-OH-3 in more human-like models, such as primary hepatocytes.

### Changes in Endogenous Metabolites Following PCB3 Exposure in HepG2 Cells

**Figure 4:**
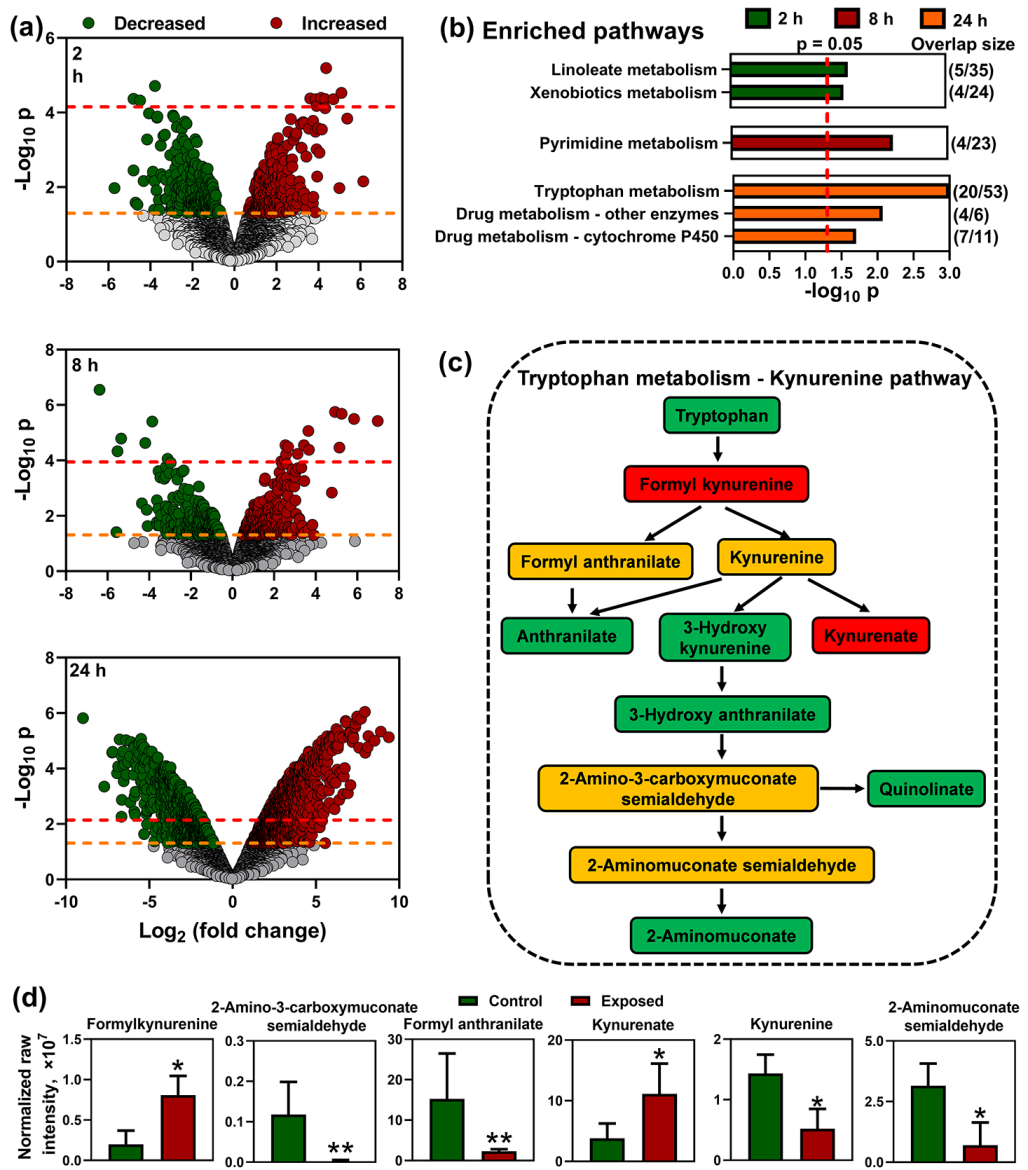
Metabolomic analysis of medium samples revealed distinct differences between experiments with HepG2 cells exposed for 2, 8, or 24 h to PCB3 and a vehicle (DMSO). (a) Volcano plots with data from 2, 8, or 24 h incubations selected 555, 534, and 1929 features using a threshold of *p* = 0.05 (yellow line) and 10, 20, and 966 features using FDR threshold = 0.05 (red line). (b) Pathway enrichment analyses with feature lists containing raw *p* values identified 2, 1, and 3 affected metabolic pathways for PCB exposures of 2, 8, and 24 h, respectively (*p* < 0.05). Pathways with less than four significant features were not presented. A metabolite was included in the pathway analysis only if the primary molecular ion ([M-H]^−^) was statistically significant between groups. The number of features altered by PCB3 exposure is listed as overlap/total features for each pathway. (c) Tryptophan metabolism was identified as significantly affected by PCB3 exposure at the 24 h time point. Metabolites with yellow, red, and green backgrounds decreased, increased, or did not change due to PCB3 exposure, respectively. Metabolites in white boxes could not be identified with acceptable confidence scores. (d) Changes in the tryptophan metabolism–kynurenine pathway following exposure of HepG2 cells to PCB3 with levels of 5-hydroxyindoleacetaldehyde, indolepyruvate, kynurenine, serotonin, 5-hydroxytryptophan, and 6-hydroxymelatonin decreasing and levels of methylserotonin, formylkynurenine, and formyl-acetyl-5-methoxykynurenamine increasing. Data are shown as normalized raw intensity, with *p* < 0.05 (*) or *p* < 0.01 (**). The accurate *m*/*z*, retention times, adducts, significances, and confidence scores of the metabolite annotations in the tryptophan metabolism pathway are listed in Table S5. For information about the pathway enrichment analyses with a looser parameter setting, see Figure S14.

We performed metabolomic analyses with the LC-Orbitrap MS data to investigate changes in endogenous metabolic pathways in HepG2 cells following PCB3 exposure. In the univariate analyses, we identified 555, 534, and 1929 metabolic features (*p* < 0.05) and 10, 20, and 966 features with a false discovery rate (FDR) < 0.05 that significantly differed between control and PCB3-exposed media at the 2, 8, and 24 h time points (Figure 4a). Metabolic pathways enriched in these significant features were identified using *mummichog* with a human pathway library. Two, one, and three metabolic pathways were significantly affected at the 2, 8, and 24 h time points (*p* < 0.05) (Figure 4b). Pathway enrichment analyses with a looser parameter setting identified an overlap in pathways affected at the 2 and 8 h but not the 24 h time point (i.e., linoleate metabolism and fatty acid metabolism, Figure S13). It is not surprising that the effects of PCB3 on the metabolome in the experimental system change over time due to adaptive responses of the cells and time-dependent changes in the PCB3 and the PCB3 metabolite mixture present in the cells. These changes reflect the effects of PCB3 on the transport or cellular metabolism of endogenous metabolites in our model system.

The kynurenine pathway of tryptophan metabolism, which accounts for most of the dietary tryptophan metabolism in the liver,^78^ was enriched at the 24 h time point, with 20 significantly changed features (Figure 4c). Briefly, formyl kynurenine and kynurenate levels increased, and 2-amino-3-carboxymuconate semialdehyde, formyl anthranilate, kynurenine, and 2-aminomuconate semialdehyde levels decreased in HepG2 cells following PCB3 exposure (Figure 4d). A link between PCB exposure and tryptophan metabolism has been observed in animal studies. For example, Aroclor 1254, a PCB mixture, can alter tryptophan metabolism by inhibiting tryptophan hydroxylase (TPH) and consequently reducing the levels of serotonin, a neurotransmitter, in the rat brain.^79^ An inhibition of TPH or a reduction of serotonin levels was also observed in aquatic organisms, such as Atlantic croaker^80,81^ and bluegill sunfish,^82^ following PCB exposure. It remains unclear how exposure to PCB3 or its metabolites causes alterations in the kynurenine pathway. Key enzymes in this pathway (e.g., kynurenine aminotransferase, kynurenine monooxygenase, and kynureninase) are vitamin B_6_-dependent.^83−86^ Because the levels of vitamin B_6_ are altered by PCB exposure in the rat liver^87^ and HepG2 cells,^46^ altered levels of vitamin B_6_ or pyridoxal phosphate (PLP), its biologically active form, may play a role in alterations of the kynurenine pathway in HepG2 cells following PCB exposure. Unfortunately, we were unable to identify PLP in this study. Targeted metabolome screens are warranted to determine how lower chlorinated PCBs and their metabolites affect hepatic tryptophan metabolism, especially at concentrations and dosing paradigms that reflect current human exposures to PCB3.

### Metabolome-Wide Association Study with PCB3 Metabolite Classes

A metabolome-wide association study revealed endogenous metabolic features positively or negatively associated with the PCB3 metabolite classes identified in our study (Figure S14). While this analysis does not identify causal relationships, it allows the development of hypotheses for subsequent experiments. Many metabolic features were associated with OH-PCB3 (1438 with *p* < 0.05; 180 with FDR < 0.05), PCB3 sulfate (1525 with *p* < 0.05; 176 with FDR < 0.05), and MeO-PCB3 sulfate metabolites (1020 with *p* < 0.05; 151 with FDR < 0.05). A smaller number of metabolic features were associated with the other PCB3 metabolite classes (45–137 with *p* < 0.05; 3–24 with FDR < 0.05). This analysis suggests that OH-PCB3, PCB3 sulfate, and MeO-PCB3 sulfate metabolites have broader effects on the metabolome in the HepG2 model system.

**Figure 5:**
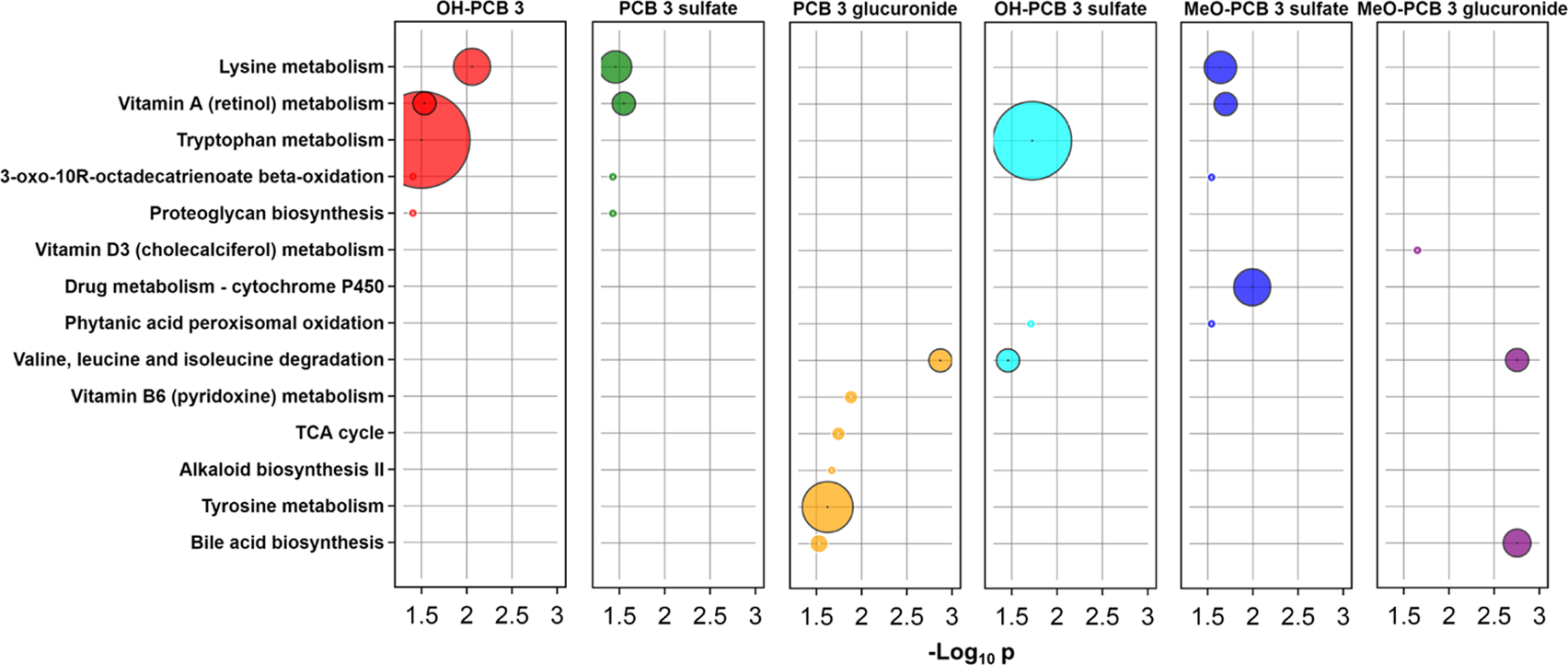
Metabolome-wide association analysis suggests that PCB3 metabolite classes formed in HepG2 cells are significantly associated with several metabolic pathways. The size of circles is proportional to the overlap size (number of significant features) of the pathway enrichment. Circles with black borders are major pathways with >5 significantly associated features. Metabolome-wide association analyses were performed on 18 samples incubated with and without PCB3. Peak areas of the PCB3 metabolites were integrated across all samples and normalized for the total intensities (extracted in the metabolomics analysis) to account for the total mass and recovery effects. Intensities were summed when more than one isomer of each PCB3 metabolite class was detected. For more details about the data analyses, see the Experimental Section. For the number of significantly associated features with each metabolite class, see Figure S13.

Pathway enrichment analyses identified several endogenous metabolic pathways that are significantly associated (*p* < 0.05) with specific PCB3 metabolite classes (Figure 5). Major pathways identified through this analysis include amino acid metabolism pathways, vitamin A (retinol) metabolism, and bile acid biosynthesis. OH-PCB3 and OH-PCB3 sulfate metabolites were associated with tryptophan metabolism, with more than 20 significant features (*p* = 0.0316 and 0.0187, respectively). The PCB3 glucuronide and MeO-PCB3 glucuronide metabolites were associated with branched-chain amino acid metabolism (*p* = 0.0013 and 0.0018, respectively). Potential co-effects of PCB3 metabolite classes on endogenous metabolic pathways are summarized in a network plot (Figure S15). Associations between the levels of PCBs or their metabolites and metabolome data have received little attention. One cohort study identified several metabolic pathways associated with PCB exposure in maternal and cord sera, including altered purine and pyrimidine metabolism and amino acid metabolism.^88^ Our results demonstrate that, in addition to the parent compounds, PCB metabolites affect the metabolome of HepG2 cells by altering the cellular uptake, metabolism, or removal of endogenous metabolites from PCB3-exposed HepG2 cells. Because the levels of PCB3 metabolites have not been characterized in the human liver, it is unclear if the associations identified in this analysis reflect actual human liver concentrations.

## Supporting Information Available

Details regarding the chemicals and materials; ADMET Predictor and MetaDrug prediction results; cell culture and cytotoxicity assays and data; GC–MS operation conditions for PCB3 analyses and data; LC–MS operation conditions for PCB3 metabolite analyses and data; metabolites data related to tryptophan metabolism; MS and MS/MS data of PCB3 metabolites; the number of endogenous metabolites associated with PCB3 metabolites classes (PDF)
