# Supplementary material for: Characterization of the Metabolic Pathways of 4-Chlorobiphenyl (PCB3) in HepG2 Cells Using the Metabolite Profiles of Its Hydroxylated Metabolites
Source: Environ Sci Technol. 2021 Jun 14;55(13):9052–62. doi: 10.1021/acs.est.1c01076 (PMC8264946; doi:10.1021/acs.est.1c01076)
Supplement: Supplementary file 1 — es1c01076_si_001.pdf [file es1c01076_si_001.pdf]

SUPPORTING INFORMATION

CHARACTERIZATION OF THE METABOLIC PATHWAYS OF 4-CHLOROBIPHENYL (PCB3) IN HepG2 CELLS USING THE METABOLITE PROFILES OF ITS HYDROXYLATED METABOLITES

Chun-Yun Zhang[1], Susanne Flor[1], Patricia Ruiz[2], Gabriele Ludewig[1], Hans-Joachim Lehmler[1,*]

[1]Department of Occupational and Environmental Health, The University of Iowa, Iowa City, Iowa 52242, United States, [2]Office of Innovation and Analytics, Simulation Science Section, Agency for Toxic Substances and Disease Registry, Atlanta, Georgia 30333, United States

Corresponding Author:
Dr. Hans-Joachim Lehmler
The University of Iowa
Department of Occupational and Environmental Health
University of Iowa Research Park, #164 MTF
Iowa City, IA 52242-5000
Phone: (319) 335-4981
Fax: (319) 335-4290
e-mail: hans-joachim-lehmler@uiowa.edu

Number of pages: 39
Number of tables: 6
Number of figures: 15

Table of Contents

Chemicals and Materials	S4
In silico metabolite predictions with ADMET Predictor and MetaDrug	S4
Cell culture	S7
Toxicity assessment through cell count	S7
Resazurin viability assay	S8
Gas chromatography-mass spectrometric (GC-MS) analysis	S8
Liquid chromatography-mass spectrometric (LC-MS) analysis	S9
Quality assurance/quality control (QA/QC)	S10
Metabolomic analysis	S11
**Table S1.** Several PCB3 metabolites were detected by LC-Orbitrap MS in medium from HepG2 cells exposed to 10 μM PCB3 for 2, 8, and 24 h	S12
**Table S2.** Metabolism of PCB3 by human cytochrome P450 enzymes and UDP-glucuronosyltransferases (UGTs) predicted by ADMET Predictor	S13
**Table S3.** Metabolites formed from PCB3 and its metabolites in humans, as predicted by MetaDrug	S16
**Table S4.** Summary of PCB3 metabolites detected by LC-QTof MS in medium from HepG2 cells exposed to 10 μM PCB3 for 2, 8, and 24 h	S18
**Table S5.** The *m/z*, retention times, p-values, and confidence levels of the metabolites in the tryptophan metabolism-kynurenine pathway identified through the metabolomic analysis for HepG2 cells exposed to PCB3 or vehicle for 24 h	S19
**Table S6.** QA/QC data for the quantification of the PCB3	S20
**Fig. S1.** The cytotoxicity of PCB3 and its hydroxylated metabolites toward HepG2 cells by the (a) cell counting method and (b) resazurin method reveals no toxicity at the concentration investigated	S21
**Fig. S2.** PCB3 concentration quantified in the cell culture medium decreased with increasing incubation time	S22
**Fig. S3** LC-Orbitrap MS data with an accurate mass of the molecular ion $[M-H]^-$, the isotope pattern consistent with a monochlorinated compound (A : A+2 = 1 : 3), and the MS/MS data support the formation of an isomer of an OH-PCB3 metabolite eluting at 5.53 min in HepG2 cells exposed to PCB3	S23
**Fig. S4.** LC-Orbitrap MS data with accurate masses of the molecular ion $[M-H]^-$, the isotope patterns consistent with a monochlorinated compound (A : A+2 = 1 : 3), and the MS/MS data support the formation of isomers of PCB3 sulfate metabolites eluting at (a) 4.20 min,(b) 4.26 min and (c) 4.30 min in HepG2 cells exposed to PCB3	S24
**Fig. S5.** LC-Orbitrap MS data with an accurate mass of the molecular ion $[M-H]^-$, the isotope pattern consistent with a monochlorinated compound (A : A+2 = 1 : 3), and	S25

the MS/MS data support the formation of an isomer of a PCB3 glucuronide metabolite eluting at 3.83 min in HepG2 cells exposed to PCB3.

Fig. S6. LC-Orbitrap MS data with accurate masses of the molecular ion $[M-H]^-$, the isotope patterns consistent with a monochlorinated compound (A : A+2 = 1 : 3), and the MS/MS data support the formation of isomers of OH-PCB3 sulfate metabolites eluting at (a) 3.50 min and (b) 4.29 min in HepG2 cells exposed to PCB3 S26

Fig. S7. LC-Orbitrap MS data with accurate masses of the molecular ion $[M-H]^-$, the isotope patterns consistent with a monochlorinated compound (A : A+2 = 1 : 3), and the MS/MS data support the formation of isomers of MeO-PCB3 sulfate metabolites eluting at (a) 4.25 min and (b) 4.29 min in HepG2 cells exposed to PCB3 S27

Fig. S8. LC-Orbitrap MS data with an accurate mass of the molecular ion $[M-H]^-$, the isotope pattern consistent with a monochlorinated compound (A : A+2 = 1 : 3), and the MS/MS data support the formation of an isomer of a MeO-PCB3 glucuronide metabolite eluting at 3.86 min in HepG2 cells exposed to PCB3 S28

Fig. S9. LC-QTof MS data with an accurate mass of the molecular ion $[M-H]^-$ and the isotope pattern consistent with a monochlorinated compound (A : A+2 = 1 : 3) support the formation of a 3'- or 4'-OH-PCB3 metabolite by HepG2 cells exposed to PCB3 S29

Fig. S10. LC-QTof MS data with accurate masses of the molecular ion $[M-H]^-$ and the isotope patterns consistent with a monochlorinated compound (A : A+2 = 1 : 3) support the formation of (a) 3-PCB3 sulfate, (b) 4-PCB2 sulfate, (c) 3'-PCB3 sulfate and (d) 4'-PCB3 sulfate by HepG2 cells exposed to PCB3 S30

Fig. S11. LC-QTof MS data with an accurate mass of the molecular ion $[M-H]^-$ and the isotope pattern consistent with a monochlorinated compound (A : A+2 = 1 : 3) support the formation of a 4'-PCB3 glucuronide metabolite by HepG2 cells exposed to PCB3 S31

Fig. S12. LC-QTof data with accurate masses of the molecular ion $[M-H]^-$ and the isotope patterns consistent with a monochlorinated compound (A : A+2 = 1 : 3) support the formation of two isomers of MeO-PCB3 sulfate metabolites eluting at (a) 8.46 min and (b) 9.05 min in HepG2 cells exposed to PCB3 S32

Fig. S13. Pathway enrichment analysis revealed several endogenous metabolic pathways were altered in HepG2 cells following PCB 3 exposure for (a) 2 h, (b) 8 h, and (c) 24 h. S33

Fig. S14. A metabolome-wide association study with six PCB3 metabolite classes suggests that OH-PCB3, PCB3 sulfate, and MeO-PCB3 sulfate have broader effects on the HepG2 cell metabolome than other metabolite classes S34

Fig. S15. Network correlation analysis revealed co-effects of PCB3 metabolite classes on the endogenous metabolic pathways S35

References S36

Chemicals and Materials. 4-Chlorobipheyl (PCB3), 4'-chloro-2-hydroxy-biphenyl (2'-OH-3), 4'-chloro-3-hydroxy-biphenyl (3'-OH-3), 4'-chloro-4-hydroxy-biphenyl (4'-OH-3), 4-Chloro-2-hydroxy-biphenyl (2-OH-3), 4-chloro-3-hydroxy-biphenyl (3-OH-3), 3-chloro-4-hydroxy-biphenyl (4-OH-2) and 4'-chloro-3,4-dihydroxy-biphenyl (3',4'-di-OH-3) used as test compounds and analytical standards of 4'-chloro-2-sulfooxy-biphenyl (2'-sulfate), 4'-chloro-3-sulfooxy-biphenyl (3'-sulfate), 4'-chloro-4-sulfooxy-biphenyl (4'-sulfate), 4'-chloro-3'-fluoro-4-sulfooxy-biphenyl (3-F,4'-PCB3 sulfate) and 4'-chloro-3'-fluoro-4-hydroxy-biphenyl (3-F,4'-OH-PCB3) were synthesized and authenticated previously.[1-4] 3,5-Dichlorobiphenyl (PCB14, surrogate standard for PCB3) and 4,4'-dichlorobiphenyl (PCB15, internal standard for PCB3) were purchased from AccuStandard, Inc. (New Haven, CT, USA).

Resazurin sodium salt was purchased from Sigma-Aldrich (St Louis, MO, USA). Phenol red-free minimum essential medium (MEM), fetal bovine serum (FBS), L-glutamine, glucose solution, penicillin/streptomycin (P/S), Dulbecco's phosphate-buffered saline (PBS), trypsin-EDTA, Costar plates, as well as dimethyl sulfoxide (DMSO) (Acros Organics), were obtained through Thermo Fisher Scientific Inc. (Radnor, PA, USA). The spin filters (4 mL, part number ECQUSF24CT) used for the sample preparation were purchased from United Chemical Technologies, Inc. (Bristol, PA, USA).

***In silico* metabolite predictions with ADMET Predictor and MetaDrug.** The *in silico* metabolite predictions were performed with ADMET Predictor (Simulations Plus, Lancaster, CA, USA.) and MetaDrug (Thompson Reuters, New York, NY, USA).[5] Briefly, structures of PCB3 and putative metabolites, including hydroxylated, sulfated, glucuronidated, and methoxylated metabolites, were drawn in Mol file format using ChemBioDraw Ultra and uploaded to both programs.[6] The structures of possible metabolites were predicted with the

default settings of the Metabolism Module of ADMET Predictor or MetaDrug to develop a subject screening list for the metabolism studies in HepG2 cells. PCB3 and its metabolites were also classified as substrates of selected cytochrome P450 isoforms (CYP1A2, CYP2A6, CYP2B6, CYP2C8, CYP2C19, CYP2C9, CYP2D6, CYP2E1, and CYP3A4 for ADMET Predictor; CYP1A2, CYP2B6, CYP2D6, and CYP3A4 for MetaDrug).

Prediction of PCB3 metabolism. A subject screening list with PCB3 metabolites likely formed in humans was developed with ADMET Predictor and MetaDrug. ADMET Predictor suggested the formation of 4'-OH-3 by CYP1A2 and CYP2D6, with minor contributions from CYP2C19 and CYP3A4 (Table S2). Similarly, ADMET Predictor indicated that CYP1A2 and CYP2D6 primarily oxidize PCB11, another low-chlorinated PCB congener, in the *para* position.[5] Experiments with purified rat cytochrome P450 enzymes confirm the role of CYP1A2 in the oxidation of PCB11 in the *para* position.[7] In contrast, *ortho*-chlorinated PCBs are metabolized by CYP2A6 and CYP2B6, but not CYP1A2 and CYP3A4.[6, 8] Therefore, experimental studies are needed to confirm that CYP1A2 and CYP2D6 metabolize lower-chlorinated PCBs.

MetaDrug suggested the formation of 3'-OH-3 and 4'-OH-3 and other PCB3 metabolites, such as PCB3 epoxides, mono-methoxylated, sulfated and glucuronidated metabolites, and catechol and other di-hydroxylated PCB3 metabolites (Table S3). Metadrug also indicated the formation of dechlorinated metabolites, including (hydroxylated) glutathione or cysteine conjugates of biphenyl. As we reported elsewhere, MetaDrug predicted similar metabolites for PCB11.[5]

Predicted metabolism of selected PCB3 metabolites. We predicted the biotransformation of PCB3 metabolites (i.e., hydroxylated, sulfate, glucuronide, and methoxylated-hydroxylated metabolites) with both programs to expand the subject screening list of likely, human-relevant

PCB3 metabolites (Tables S2-S3). ADMET Predictor predicted the cytochrome P450 enzyme-mediated formation of di- or tri-hydroxylated metabolites from mono- or di-hydroxylated PCBs, quinone metabolites from mono- and di-hydroxylated PCBs, and hydroxylated sulfate metabolites from PCB3 sulfates. PCB glucuronides were not subject to further oxidation, according to ADMET Predictor. With methoxylated and hydroxylated PCB3 metabolites as starting compounds, ADMET Predictor suggested the formation of demethylated metabolites (i.e., di-hydroxylated metabolites) and the corresponding PCB quinones. CYP1A2 and CYP2C9 were identified as cytochrome P450 isoforms likely involved in the oxidation of mono- and di-hydroxylated PCB3 metabolites, whereas only CYP1A2 contributed to the oxidation of PCB3 sulfates. ADMET Predictor also indicated that hydroxylated PCB3 metabolites are glucuronidated by UGTs.

MetaDrug predicted that the metabolism of mono- and di-hydroxylated metabolites and PCB3 sulfate is regioisomer dependent. Briefly, the metabolites profile indicated for 4'-OH-3 is more like that of PCB3. Only a di-hydroxylated metabolite and the corresponding quinone metabolite were predicted for the metabolism of 3'-OH-3. According to MetaDrug, 3-OH-3 and 4-OH-2 form monohydroxylated methyl ether, sulfate and glucuronide metabolites, and dihydroxylated cysteine and glutathione adducts. Starting with PCB3 sulfate, MetaDrug predicted the formation of PCB di-sulfate, glucuronidated sulfates, methoxylated sulfates, and cysteine or glutathione adducted sulfate metabolites with or without the addition of a hydroxyl group. The glucuronide metabolite was predicted to be dehydrogenated on the glucuronide moiety without the formation of other metabolites. Starting with the 3',4'-catechol metabolite, the software suggested the formation of the corresponding quinone metabolite and hydroxylated quinone metabolites. These predictions are consistent with *in vitro* studies demonstrating the oxidation of di-hydroxylated

PCBs to quinones.[9, 10] Moreover, we tentatively identified hydroxylated PCBs quinones of lower chlorinated PCBs in feces from mice exposed orally to an environmental PCB mixture.[11] Consistent with ADMET Predictor, MetaDrug predicted the formation of demethylated metabolites and the corresponding quinones for the methoxylated and hydroxylated PCB3 metabolites.

Cell culture. HepG2 cells were purchased from American Type Culture Collection (ATCC) (Manassas, VA, USA). The authenticity of the human hepatocellular carcinoma cell line HepG2 was confirmed by analysis of genomic DNA conducted by the University of Arizona Genetics Core (Arizona Research laboratories, Tucson, AZ, USA). The HepG2 cells used in this study were between passages 18 through 35. As described previously, cells were maintained in complete medium (MEM supplemented with 10 % FBS, 100 U/mL penicillin, 100 μg/mL streptomycin, and 2 mM l-glutamine) in a humidified incubator with 5% CO_2 at 37 °C.[5, 12] Exposure medium contained MEM without FBS but was supplemented with 4.5 mM D-glucose as an energy source, 100 U/mL penicillin, 100 μg/mL streptomycin, and 2 mM l-glutamine. PCB3 and its hydroxylated metabolites were dissolved in DMSO. The final concentration of DMSO in the medium did not exceed 0.1 % (v/v). This DMSO concentration did not have any effect on cell viability, as reported previously.[5, 12]

Toxicity assessment through cell count. The total cell number was analyzed by cell count using a flow cytometer with direct volume measurement. For exposure, HepG2 cells (1.2×10^6/well) in complete MEM medium (0.6 mL) were seeded into 24-well plates. After 48 h attachment, the medium was exchanged with exposure medium (no FBS, 0.6 mL per well) containing 10 μM PCB3 and its hydroxylated metabolites (i.e., 2'-OH-3, 3'-OH-3, 4'-OH-3, 2-OH-3, 3-OH-3, 4-OH-2, or 3',4'-di-OH-3) (0.1 % DMSO). After 24 h exposure, cells were

trypsinized, resuspended as a single cell suspension in complete MEM, and analyzed using an Accuri C6 flow cytometer (BD, San Jose, CA). The assay was performed in duplicates and repeated at least 3-times. The total count of cells was plotted as percent of control (Fig. S2a).

Resazurin viability assay. This assay was performed to assess the metabolic activity of HepG2 cells after exposure to PCB3 and its hydroxylated metabolites, as described above. Viable and metabolically active cells can reduce the indicator dye resazurin to a highly fluorescent resorufin. Briefly, HepG2 cells were seeded and treated with 10 μM PCB3 and its hydroxylated metabolites in DMSO. After 24 h exposure, cells were washed once with PBS and incubated with resazurin (50 μM) in complete medium for 45 min. After incubation, fluorescence was measured with a GENios Pro microtiter plate reader (Tecan, Switzerland) using an excitation wavelength of 535 nm and an emission wavelength of 590 nm. The assay was performed in duplicates at least 3-times, and the resulting fluorescence values were then plotted as percent of control (Fig. S2b).

Gas chromatography-mass spectrometric (GC-MS) analysis. PCB3 levels were measured in the acetonitrile extracts on an Agilent 7890A gas chromatograph equipped with an SLB-5ms capillary column (30 m length, 250 μm inner diameter, 0.25 μm film thickness; Supelco, St Louis, MO, U.S.A.) and an Agilent 5975C system with a triple-axis mass selective detector (M.S.D.) and an electron ionization source, as described.[13] Analyses were performed in the selected ion monitoring (SIM) mode at *m/z* 188, 190 for PCB3, and *m/z* 222, 224 for PCB14 and PCB15 with a collision energy of 70 eV. Helium was used as the carrier gas at a constant flow rate of 1 mL/min. The oven temperature program was as follows: 50 °C starting temperature, hold for 1 min, 15 °C/min to 175 °C, hold for 14 min, 15 °C/min to 280 °C, and hold for 3 min.

The transfer line temperature was 280 °C. The temperatures of the MS source and quadrupole were 230 °C and 150 °C, respectively.

Liquid chromatography-mass spectrometric (LC-MS) analysis. An initial screening for PCB3 metabolites was performed on an ultra-performance liquid chromatograph (UPLC) (Waters Acquity UPLC, Milford, MA, USA) coupled with Quadrupole Time-of-flight Mass Spectrometer (QTof MS; Waters Q-Tof Premier, Milford, MA, USA) at the High-Resolution Mass Spectrometry Facility of the University of Iowa (Iowa City, IA, USA). A Waters Acquity BEH C-18 column (2.1 mm inner diameter, 100 mm length, 1.7 µM particle size; Waters) was used for the chromatographic separation with a flow rate of 0.2 mL/min. The mobile phase was (A) water with 0.04 % (v/v) triethylammonium and (B) acetonitrile. The following solvent gradient (% (B)) was used: 0-1 min, 15%; 1-3 min, 15-30 %; 3-10 min, 30-40 %; 10-16 min, 40-60 %, 16-16.1 min, 60-95 %, 16.1-20 min, 95 %. Full scans were performed in the ESI^- mode with mass to charge ratios (*m/z*) ranging from 75 to 800 Da at a rate of 0.2 s/scan. Leucine enkephalin was infused (10 µL/min) as the lock mass and analyzed separately in the ESI^- mode. The sampling cone voltage was 35 V. The desolvation gas was operated at 350 °C with a flow rate of 650 L/h. The capillary voltage was 2.8 kV.

Media samples were subsequently analyzed with a UPLC (Ultimate 3000 UHPLC+ Focused, Thermo Fisher, Waltham, MA, USA) coupled with a Q Exactive Hybrid Quadrupole-Orbitrap mass spectrometer (LC-Orbitrap MS; Thermo Fisher) at the Center of Mass Spectrometry and Proteomics at the University of Minnesota (Minneapolis, MN, USA) using full scan and MS/MS methods. The metabolites from experiments with PCB3 were separated with an Acquity UPLC BEH C-18 column (2.1 mm inner diameter, 100 mm length, 1.7 µM particle size; Waters).[5] The flow rate was 0.3 mL/min. The following gradient (% (B) acetonitrile) was used: 0-1 min, 15 %;

1-3 min, 15-60 %; 3-11.5 min, 60-90 %; 11.5-12 min, 90-98 %; 12-12.5 min, 98%; 12.5-13 min 98-15 %; and 13-14 min, 15 %. Water with 10 mM ammonium acetate (pH = 7.0) was used as solvent (A). The Orbitrap MS system was equipped with heated-electrospray ionization (HESI-Ⅱ) probe source and operated in the negative mode using the following conditions: Spray voltage, 3.0 kV; sheath gas flow rate, 50 arb; capillary temperature, 320 °C; auxiliary gas heater, 400 °C; S-lens, 55 V. Full-scan accurate mass spectra were obtained in a scan range from *m/z* 70 to *m/z* 1050 with a resolution of 70000 FWHM. Targeted MS/MS spectra for PCB3 metabolites and data-dependent MS/MS spectra for endogenous metabolites were collected at a collision energy of 30 eV. Since no PCB metabolites were detected in the ESI positive mode in our earlier studies,[5, 12] only ESI negative data were collected and processed.

Quality assurance/quality control (QA/QC). QA/QC data for the quantification of PCB3 in cell culture media were listed in Table S6. QA/QC data for PCB3 metabolite analyses were as follows: Extracted ion chromatograms are based on calculated exact masses with a mass window of 10 ppm. PCB3 metabolites are only reported if they were detected in all triplicate samples with an abundance of >10-times of the background levels. The following criteria were used to identify PCB3 metabolites: Differences between measured and calculated accurate mass were < 5 ppm. The isotopic mass patterns of the molecular ions were consistent with the pattern predicted for PCB3 metabolites within a 20 % error. MS/MS spectra were acquired for at least one isomer of each PCB3 metabolite class whenever possible to confirm structural elements. 3-F-4'-OH-PCB3 and 3-F-4'-PCB3 sulfate were used to monitor the performance of the extraction and instrumental analysis. Control samples, including method blanks (no matrix), medium blanks (not incubated with cells), and HepG2 cells incubated with DMSO, were analyzed in parallel. No PCB metabolites were detected in any control sample. Control samples also include cell culture

media (no cells) incubated with 3',4'-di-OH-3 (a likely less-stable compound). No further transformation products were detected in these control samples.

Metabolomic analysis. The raw LC-Orbitrap MS data were extracted using *apLCMS*[14] and *xMSanalyzer*[15] to obtain a peak table with mass to charge ratio (*m/z*), retention times, and intensities for each mass spectral feature. Features were only included in further analyses when they were detected in at least two replicates from the same experimental group. The missing values were imputed with one-half of the lowest signal detected for that feature across all samples. The data were $\log_2$ transformed and normalized by the sum of the peak areas of all the ions. Changes in metabolic features were identified using limma[16] for generating raw p-values. p-Values adjusted for false discovery rate (FDR) in multiple comparisons were obtained using the Benjamini and Hochberg method.[17] Volcano plots of the $-\log_{10}$ p-values versus $\log_2$ fold change were used to visualize the feature distribution (raw p-values ≤ 0.05; q-values ≤ 0.05 for FDR correction). Pathway enrichment analysis using *mummichog* (version 2.1.1)[18] was performed on the raw p-values. *Mummichog* provides a biological interpretation of the data without a priori identification of the metabolites in the untargeted feature table. A less stringent criterion of raw $p < 0.05$ was used in the pathway analysis to obtain optimal coverage of metabolites (100-500 metabolites). This approach protects against type 1 statistical error by permutation testing in the pathway enrichment analysis in addition to the *limma* test.[16] All the metabolites were annotated using *xMSannotator*[19] with confidence scores based on the Human Metabolome DataBase (HMDB)[20] and the Kyoto Encyclopedia of Genes and Genomes (KEGG),[21] as described.[5] Alternatively, the metabolites were identified with experimental MS/MS spectra using *metID*[22] based on public MS/MS databases, including HMDB[20] and MassBank.[23]

Table S1. Several PCB3 metabolites were detected by LC-Orbitrap MS in medium from HepG2 cells exposed to 10 μM PCB3 for 2, 8, and 24 h.[a]

Metabolites	Retention time[b], min	Formula	Normalized intensity[c]			Accurate mass difference[d], ppm	MS^2 (Da)	Confidence level[e]
			2 h	8 h	24 h			
OH-PCB3	5.53	$C_{12}H_8ClO^-$	0.53±0.09	14±2	22±5	3.63	174.99, 167.05	2
PCB3 sulfate or PCB 2 sulfate	4.20	$C_{12}H_8ClSO_4^-$	0.88±0.01	22±1	28±4	2.72	203.03	2
	4.26		0.39±0.03	6.8±0.9	9±1	2.86	203.03	2
	4.30		1.16±0.04	39±3	70±5	2.79	203.03	2
PCB3 glucuronide	3.83	$C_{18}H_{16}ClO_7^-$	ND	0.72±0.03	4.9±0.3	2.72	203.03	2
OH-PCB3 sulfate	3.50	$C_{12}H_8ClSO_5^-$	ND	0.36±0.04	1.3±0.1	2.78	219.02	3
	4.29		ND	6.6±0.5	4.3±0.3	2.74		2
MeO-PCB3 sulfate	4.25	$C_{13}H_{10}ClSO_5^-$	ND	4.0±0.4	18±1	3.16	233.04, 218.01	2
	4.29		ND	7.0±0.4	31±1	3.13	233.04, 218.01	2
MeO-PCB3 glucuronide	3.86	$C_{19}H_{18}ClO_8^-$	ND	ND	0.31±0.02	2.64	233.04, 218.01	2

[a] HepG2 cells were exposed for 2, 8, and 24 h to 10 μM PCB3 as described in the Experimental Section, metabolites were extracted from the cell culture medium by QuEChERS extraction, and extracts were analyzed by LC-Orbitrap MS. Four Classes of PCB3 metabolites were also detected in LC-QTof analyses of the same extracts (Table S4). The corresponding MS and MS^2 spectra are provided in Figs. S3-S8.

[b] Injections for both LC-MS and MS/MS analysis were performed on an LC-Orbitrap MS with an Acquity UPLC BEH C18 column.

[c] 3-F,4'-PCB3 sulfate (100 ng) was spiked into all samples and used for the normalization. The normalized intensity is semi-quantitatively provided as the signal intensity of the metabolite peak area/internal standard peak area × 100.

[d] The accurate mass difference in parts per million (ppm) was calculated as (measured mass-calculated mass)/calculated mass × 10^6.

[e] Confidence levels for the identification of PCB metabolites were assigned using the Schymanski framework.[24] Level 1: metabolites were not only identified based on accurate mass, isotope pattern, MS, and MS^2, but also with authentic standards. Level 2: metabolites were identified based on accurate mass, isotope pattern, MS, and MS/MS. Level 3: metabolites were identified based on accurate mass, isotope pattern, and MS, but not MS/MS.

ND, not detected.

Table S2. Metabolism of PCB3 by human cytochrome P450 enzymes and UDP-glucuronosyltransferases (UGTs) predicted by ADMET Predictor.

Starting compounds	P450 isoforms	Hydroxylated metabolites	Quinone metabolites	UGT isoforms	Glucuronide metabolites
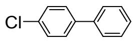	1A2 (15.2)<br>2A6<br>2B6<br>2C19 (0.1)<br>2C8<br>2D6 (1.1)<br>2E1<br>3A4 (<0.1)	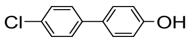			
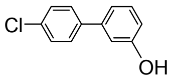	1A2 (25.0)<br>2C19 (0.4)<br>2C9 (30.9)<br>2D6 (0.1)	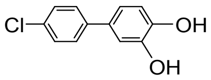	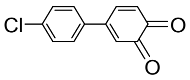	1A3<br>1A6<br>1A8<br>1A9<br>1A10<br>2B7<br>2B15	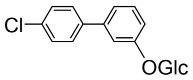
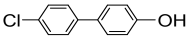	1A2 (17.7)	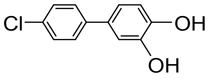	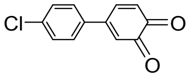	1A1<br>1A3<br>1A6<br>1A8<br>1A9<br>1A10<br>2B7<br>2B15	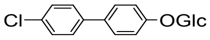
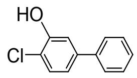	1A2 (23.6)<br>2C19 (0.4)<br>2C9 (27.2)<br>2D6 (0.6)	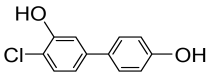		1A3<br>1A6<br>1A8<br>1A9<br>1A10<br>2B7<br>2B15	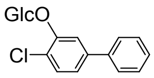
	1A2 (23.5)	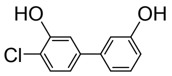			

Glc: Glucuronide; deGlc: Dehydrogenated glucuronide. For details about the metabolism prediction using ADMET Predictor, see the experimental section above.

Table S2 (continued). Metabolism of PCB3 by human cytochrome P450 enzymes and UDP-glucuronosyltransferases (UGTs) predicted by ADMET Predictor.

Starting compounds	P450 isoforms	Hydroxylated metabolites	Quinone metabolites	UGT isoforms	Glucuronide metabolites
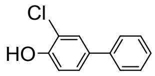	1A2 (28.9)<br>2C19 (0.3)<br>2C9 (31.9)<br>2D6 (0.7)	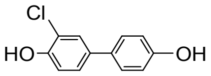		1A1<br>1A3<br>1A6<br>1A8<br>1A9<br>1A10<br>2B7<br>2B15	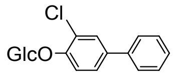
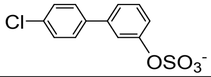	2C19 (<0.1)<br>2C9 (32.7)	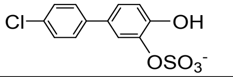			
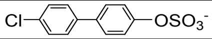					
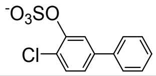	2C19 (0.1)<br>2C9 (38.0)	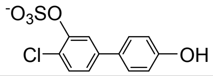			
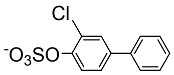	2C19 (<0.1)<br>2C9 (49.0)	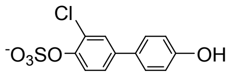			
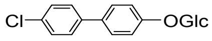	2C9	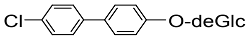		1A3<br>2B7	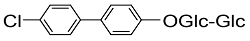
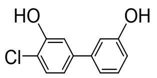	1A2 (12.4)<br>2C19 (1.4)<br>2C9 (50.4)	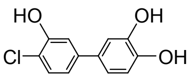	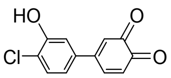	1A3<br>1A6<br>1A8<br>1A9<br>1A10<br>2B7<br>2B15	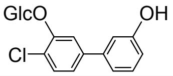<br>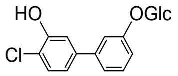
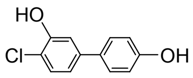	1A2 (9.6)	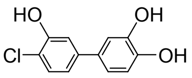	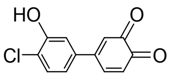	1A1<br>1A3<br>1A6<br>1A8<br>1A9<br>1A10<br>2B7<br>2B15	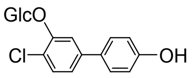

Glc: Glucuronide; deGlc: Dehydrogenated glucuronide. For details about the metabolism prediction using ADMET Predictor, see the experimental section above.

Table S2 (continued). Metabolism of PCB3 by human cytochrome P450 enzymes and UDP-glucuronosyltransferases (UGTs) predicted by ADMET Predictor.

Starting compounds	P450 isoforms	Hydroxylated metabolites	Quinone metabolites	UGT isoforms	Glucuronide metabolites
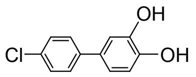			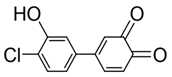	1A1<br>1A3<br>1A6<br>1A8<br>1A9<br>1A10<br>2B7<br>2B15	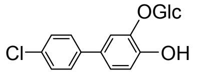<br>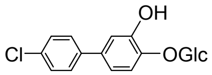
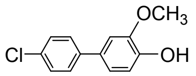	1A2 (113.1)<br>2C19 (1.2)<br>2C9 (55.3)<br>2D6 (0.1)	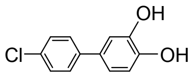	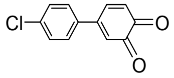	1A1<br>1A3<br>1A8<br>1A9<br>1A10<br>2B7<br>2B15	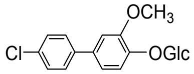
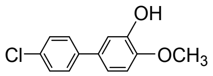	1A2 (118.8)<br>2C19 (2.0)<br>2C9 (75.1)<br>2D6 (0.1)	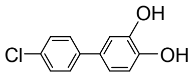	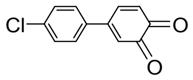	1A1<br>1A3<br>1A8<br>1A9<br>1A10<br>2B7<br>2B15	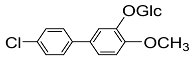

Glc: Glucuronide; deGlc: Dehydrogenated glucuronide. For details about the metabolism prediction using ADMET Predictor, see the experimental section above.

Table S3. Metabolites formed from PCB3 and its metabolites in humans, as predicted by MetaDrug.

Metabolites classes[a]	Parent compounds (abbreviations and the corresponding structures)														
	PCB 3	3'-OH-3	4'-OH-3	3-OH-3	4-OH-2	3'-PCB3 sulfate	4'-PCB3 sulfate	3-PCB3 sulfate	4-PCB2 sulfate	4'-PCB3 glucuronide	3,3'-di-OH-3	3,4'-di-OH-3	3',4'-di-OH-3	3'-MeO-4'-OH-PCB3	4'-MeO-3'-OH-PCB3
	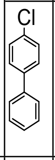	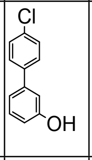	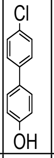	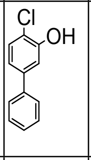	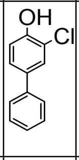	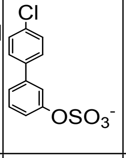	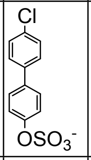	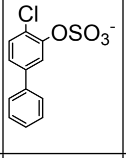	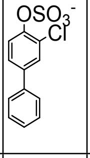	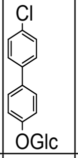	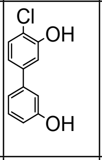	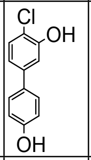	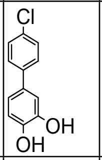	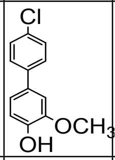	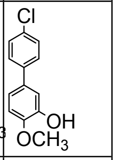
Mono-hydroxy	+														
Sulfate	+		+												
Glucuronide	+		+												
Cysteine	+														
Glutathione	+														
Di-hydroxy	+	+	+	+	+									+	+
Epoxide	+														
Monohydroxy-cysteine	+		+												
Monohydroxy-glutathione	+		+												
Methoxy	+		+												
Quinone		+											+	+	+
Tri-hydroxy			+	+	+						+				
Monohydroxy-sulfate				+	+	+	+	+	+						
Monohydroxy-glucuronide				+	+										
Dihydroxy-cysteine				+	+										
Dihydroxy-glutathione				+	+										

[a] Metabolite classes formed according to MetaDrug from PCB3 or its metabolites are indicated by "+". Only the functional group introduced into the respective parent compound, but not their position on the biphenyl moiety, are shown. The functional groups can be attached to the parent structure of PCB3 or, in the case of 4-OH-PCB2 and 4-PCB2 sulfate, PCB2.
Sul: Sulfate; Glc: Glucuronide

Table S3 (continued). Metabolism of PCB3 and its metabolites in humans predicted by MetaDrug.

Metabolites classes[a]	Parent compounds (abbreviations and the corresponding structures)														
	PCB 3	3'-OH-3	4'-OH-3	3-OH-3	4-OH-2	3'-PCB3 sulfate	4'-PCB3 sulfate	3-PCB3 sulfate	4-PCB2 sulfate	4'-PCB3 glucuronide	3,3'-di-OH-3	3,4'-di-OH-3	3',4'-di-OH-3	3'-MeO-4'-OH-PCB3	4'-MeO-3'-OH-PCB3
	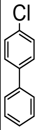	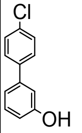	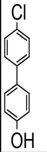	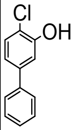	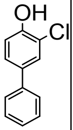	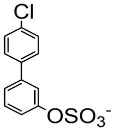	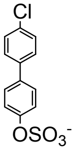	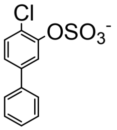	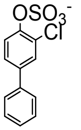	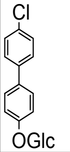	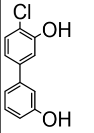	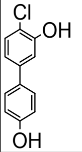	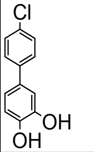	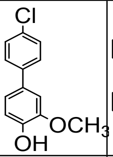	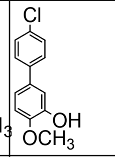
Methoxy-hydroxy				+	+										
Hydroxy-epoxide			+												
Dihydroxy-sulfate						+	+	+	+						
Hydroxy-cysteine-sulfate						+		+	+						
Hydroxy-glutathione-sulfate						+		+	+						
Sulfate-glucuronide						+		+	+						
Methoxy-sulfate						+			+						
Di-sulfate						+		+	+						
Epoxide-sulfates							+	+							
Cysteine-sulfates							+	+							
Glutathione-sulfates							+	+							
Dehydrogenated glucuronide										+					
Hydroxy-quinones											+		+		

[a] Metabolite classes formed according to MetaDrug from PCB3 or its metabolites are indicated by "+". Only the functional group introduced into the respective parent compound, but not their position on the biphenyl moiety, are shown. The functional groups can be attached to the parent structure of PCB3 or, in the case of 4-OH-2 and 4-PCB2 sulfate, PCB2.

Sul: Sulfate; Glc: Glucuronide

Table S4. Summary of PCB3 metabolites detected by LC-QTof MS in medium from HepG2 cells exposed to 10 µM PCB3 for 2, 8, and 24 h.[a]

Metabolites	Retention time[a], min	Formula	$[M-H]^-$			Confidence level[b]
			Calculated (Da)	Measured (Da)	Differences (ppm)	
3' or 4'-OH-PCB3	15.95	$C_{12}H_7Cl_2O^-$	203.0264	203.0276	6	1
3-PCB3 sulfate	7.92	$C_{12}H_7Cl_2O_4S^-$	282.9832	282.9889	20	2
4-PCB2 sulfate	8.38			282.9928	34	2
3'-PCB3 sulfate	8.76			282.9944	40	1
4'-PCB3 sulfate	9.19			282.9871	14	1
4'-PCB3 glucuronide	5.51	$C_{18}H_{16}ClO_7^-$	379.0585	379.0630	12	3
3'-MeO-4'-PCB3 sulfate or 4'-MeO-3″-PCB3 sulfate	8.46	$C_{13}H_{10}ClSO_5^-$	312.9938	312.9977	12	3
	9.05			312.9992	17	3

[a] Sample extracts of the media from PCB3-exposed and control incubations (DMSO only) were analyzed by LC-QTof-MS. A Waters Acquity BEH C-18 column (2.1 mm inner diameter, 100 mm length, 1.7 µM particle size; Waters) was used for the chromatographic separation of the PCB3 metabolites with a flow rate of 0.2 mL/min. For a detailed description of the LC-QTof-MS analyses, see Experimental Section.

[b] Confidence levels were assigned using the Schymanski framework.[24] Level 1: metabolites were not only identified based on their accurate mass, isotope pattern, MS, and MS/MS data, but also with an authentic standard. Level 2: metabolites were identified based on their accurate mass, isotope pattern, MS, and MS/MS data. Level 3: metabolites were identified based on their accurate mass, isotope pattern, and MS, but not MS/MS data.

Table S5. The *m/z*, retention times, p-values, and confidence levels of the metabolites in the tryptophan metabolism-kynurenine pathway identified through the metabolomic analysis for HepG2 cells exposed to PCB3 or vehicle for 24 h (see Fig. 4).

Metabolites[a]	Molecular ion	*m/z*	Retention time [s]	p-value[b]	Confidence level[c]
Tryptophan	$[M-H]^-$	203.0826	75.0	0.2371	3
Formyl Kynurenine	$[M-H]^-$	235.0722	64.0	0.0255	2
Formyl anthranilate	$[M-H]^-$	164.0354	71.4	0.0063	2
Kynurenine	$[M-H]^-$	207.0776	64.5	0.0166	2
Anthranilate	$[M-H]^-$	134.0403	74.1	0.9854	2
Hydroxy kynurenine	$[M-H]^-$	205.0619	128.9	0.3680	2
kynurenate	$[M-H]^-$	188.0354	67.2	0.0454	2
Hydroxy anthranilate	$[M-H]^-$	152.0354	255.4	0.9814	2
Aminocarboxymuconate semialdehyde	$[M-H]^-$	184.0248	42.8	0.0021	2
Quinolinate	$[M-H]^-$	166.0144	119.2	0.1750	2
Aminomuconate semialdehyde	$[M-H]^-$	140.0354	191.7	0.0270	2
Aminomuconate	$[M-H]^-$	156.0303	43.8	0.5173	2

[a] The metabolites were putatively annotated with xMSannotator based on the Human Metabolome DataBase (HMDB)[20] and the Kyoto Encyclopedia of Genes and Genomes (KEGG).[21] Tryptophan was also identified with experimental MS/MS spectra using *metID*[22] based on public MS/MS databases from HMDB[20] and MassBank.[23]

[b] The *p* values were obtained from *limma* test[16] on the normalized raw intensities of primary molecular ion $[M-H]^-$ between exposure and control groups incubated for 24 h.

[c] The confidence levels were assigned by the *xMSannotator* software mainly based on the number of adduct ions and isotopic ions detected for the molecular ions. Level 3 and level 2 represent high and medium confidence, respectively. Only those features annotated with high or medium confidence levels are listed.[19]

Table S6. QA/QC data for the quantification of the PCB3.

Items	Numbers of samples	Values
Linear range[a] [ng/mL]	8	1-1000
LODs[b] [ng]	3	1.2
Background levels[c] [μM]	6	0.04 (±0.01)
Recoveries of PCB14[d] [%]	18	113 (±9)

[a] The linear coefficients (R^2) of the calibration curves in a concentration range from 1 to 1000 ng/mL ranged from 0.998 to 0.999 for all the PCBs, including the surrogate standard (PCB14) and internal standard (PCB15).

[b] The limits of detection (LOD) of PCBs were calculated from method blanks as LOD = mean of blanks + 3 × standard deviation blanks.[25, 26]

[c] The background levels in PCB3 media were calculated from all control samples incubated in parallel with PCB3 exposed samples. These levels reflect the noise of the baseline in the presence of the matrix.

[d] Recoveries of the surrogate recovery standard (PCB14) from the media samples.

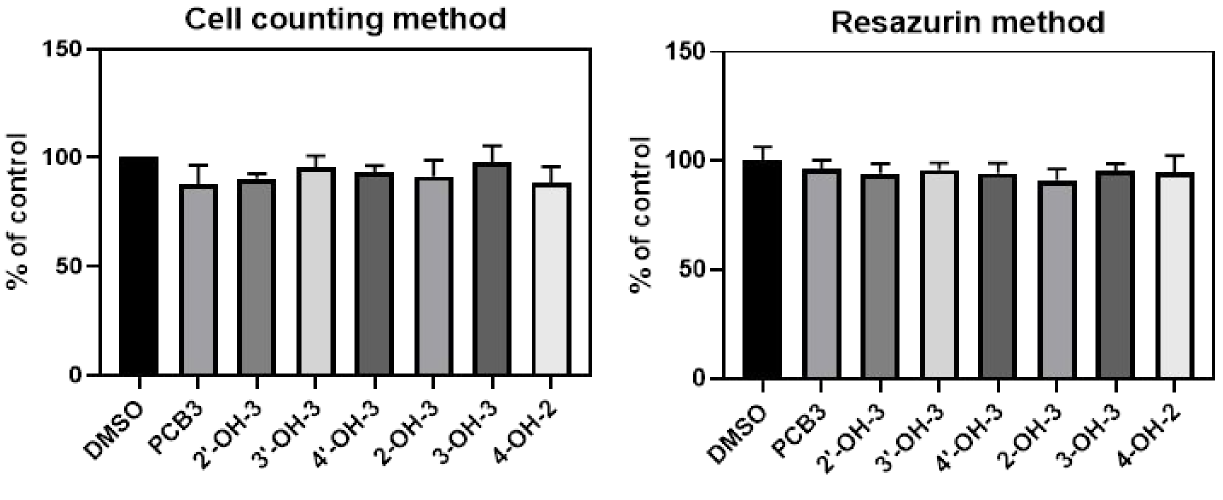

Fig. S1. The cytotoxicity of PCB3 and its hydroxylated metabolites toward HepG2 cells by the (a) cell counting method and (b) resazurin method reveals no toxicity at the concentration investigated. Briefly, HepG2 cells in 24-well plates with serum-free MEM were exposed to 10 µM PCB3 or its hydroxylated metabolites (0.1 % DMSO final concentration) for 24 h. Then cells were either detached and counted by Flow Cytometry (cell count) or incubated with resazurin (50 µM) in complete medium for 45 min, after which fluorescence was measured with a microplate reader (resazurin assay). The assay was performed in duplicates at least 3-times, and the results are plotted as percent of control. Data are given as means ± standard deviation (SD) of the independent experiments. Differences in cell viability across the control and exposure groups were analyzed using a one-way ANOVA followed by Dunnett's multiple comparisons test. For the full names of the hydroxylated PCB3 metabolites shown in this figure, see the text above.

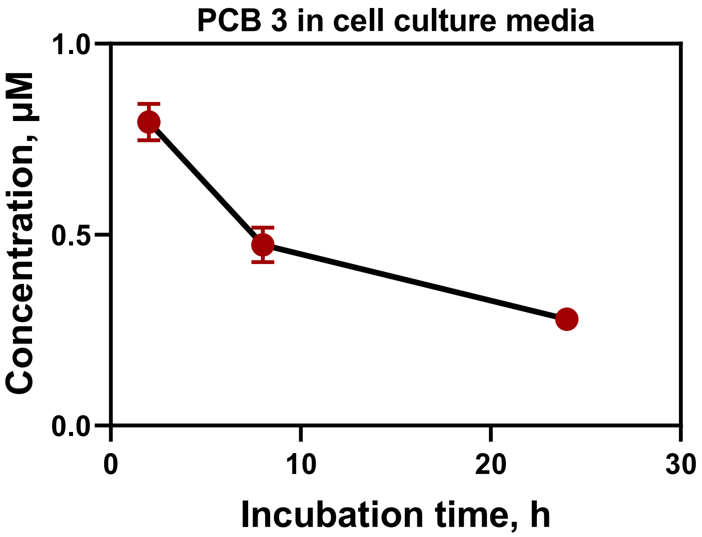

Fig. S2. PCB3 concentration quantified in the cell culture medium decreased with increasing incubation time. HepG2 cells (6×10^6/well) were seeded into 6-well plates with complete MEM medium (3 mL) per well and allowed to attach for 48 h. Cells were exposed for 2, 8, or 24 h to PCB3 (10 µM; 0.1 % DMSO) in exposure medium (3 mL). After the incubation, the cell culture media were collected, and PCB levels in media were determined with a GC-MS equipped with an SLB-5ms capillary column (30 m length, 250 µm inner diameter, 0.25 µm film thickness; Supelco, St Louis, MO, U.S.A.) and an Agilent 5975C system with a triple-axis mass selective detector and an electron ionization source. For more details, see the text above.

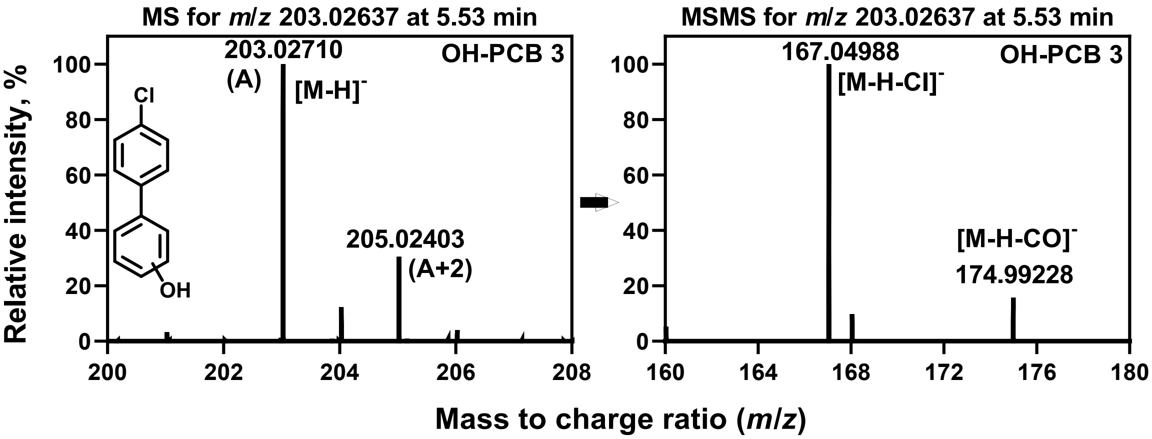

Fig. S3 LC-Orbitrap MS data with an accurate mass of the molecular ion $[M-H]^-$, the isotope pattern consistent with a monochlorinated compound (A : A+2 = 1 : 3), and the MS/MS data support the formation of an isomer of an OH-PCB3 metabolite eluting at 5.53 min in HepG2 cells exposed to PCB3. The MS/MS spectra showed featured characteristic fragment ions consistent with an OH-PCB3 (i.e., loss of CO and Cl). The placement of the functional groups on phenyl rings is for illustration purposes only and does not indicate their actual position. The collision energies on the fragmentation cell for the MS/MS spectra was 30 eV; for additional details, see the text above.

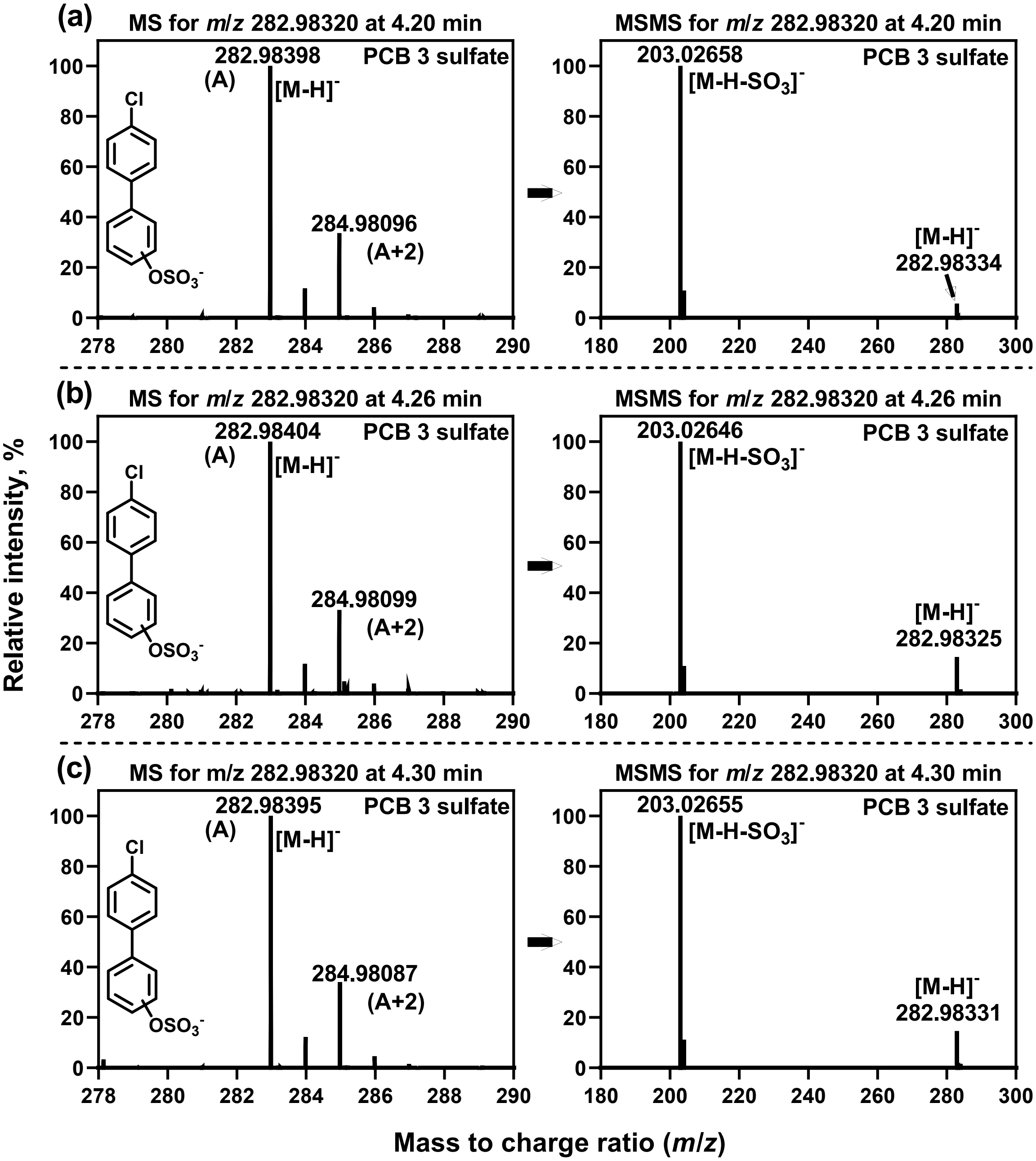

Fig. S4. LC-Orbitrap MS data with accurate masses of the molecular ion [M-H]-, the isotope patterns consistent with a monochlorinated compound (A : A+2 = 1 : 3), and the MS/MS data support the formation of isomers of PCB3 sulfate metabolites eluting at (a) 4.20 min,(b) 4.26 min and (c) 4.30 min in HepG2 cells exposed to PCB3. The MS/MS spectra showed characteristic fragment ions consistent with PCB3 sulfates (i.e., loss of SO_3). The placement of the functional groups on phenyl rings is for illustration purposes only and does not indicate their actual position. The collision energies on the fragmentation cell for the MS/MS spectra was 30 eV; for additional details, see the text above.

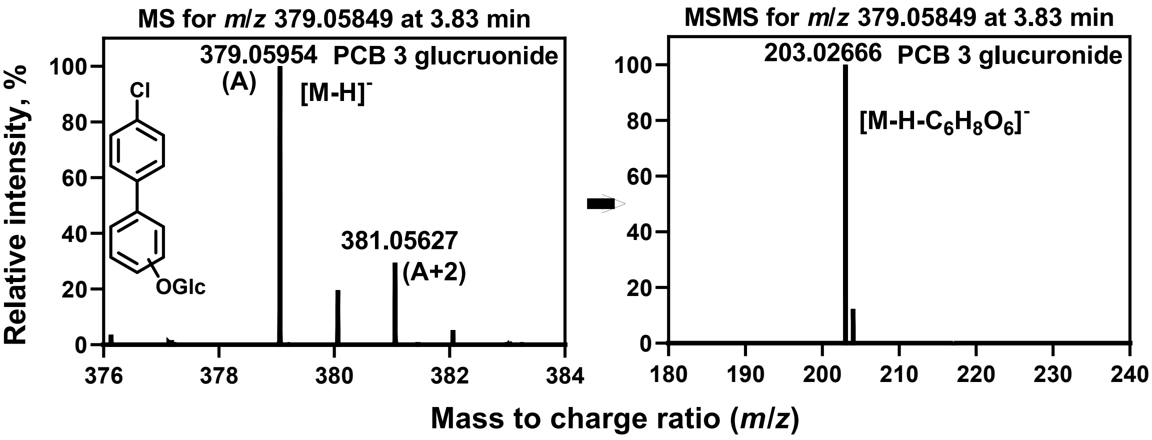

Fig. S5. LC-Orbitrap MS data with an accurate mass of the molecular ion $[M-H]^-$, the isotope pattern consistent with a monochlorinated compound (A : A+2 = 1 : 3), and the MS/MS data support the formation of an isomer of a PCB3 glucuronide metabolite eluting at 3.83 min in HepG2 cells exposed to PCB3. The MS/MS spectra showed featured characteristic fragment ions consistent with a PCB3 glucuronide (i.e., loss of $C_6H_8O_6$). The placement of the functional groups on phenyl rings is for illustration purposes only and does not indicate their actual position. The collision energies on the fragmentation cell for the MS/MS spectra was 30 eV; for additional details, see the text above.

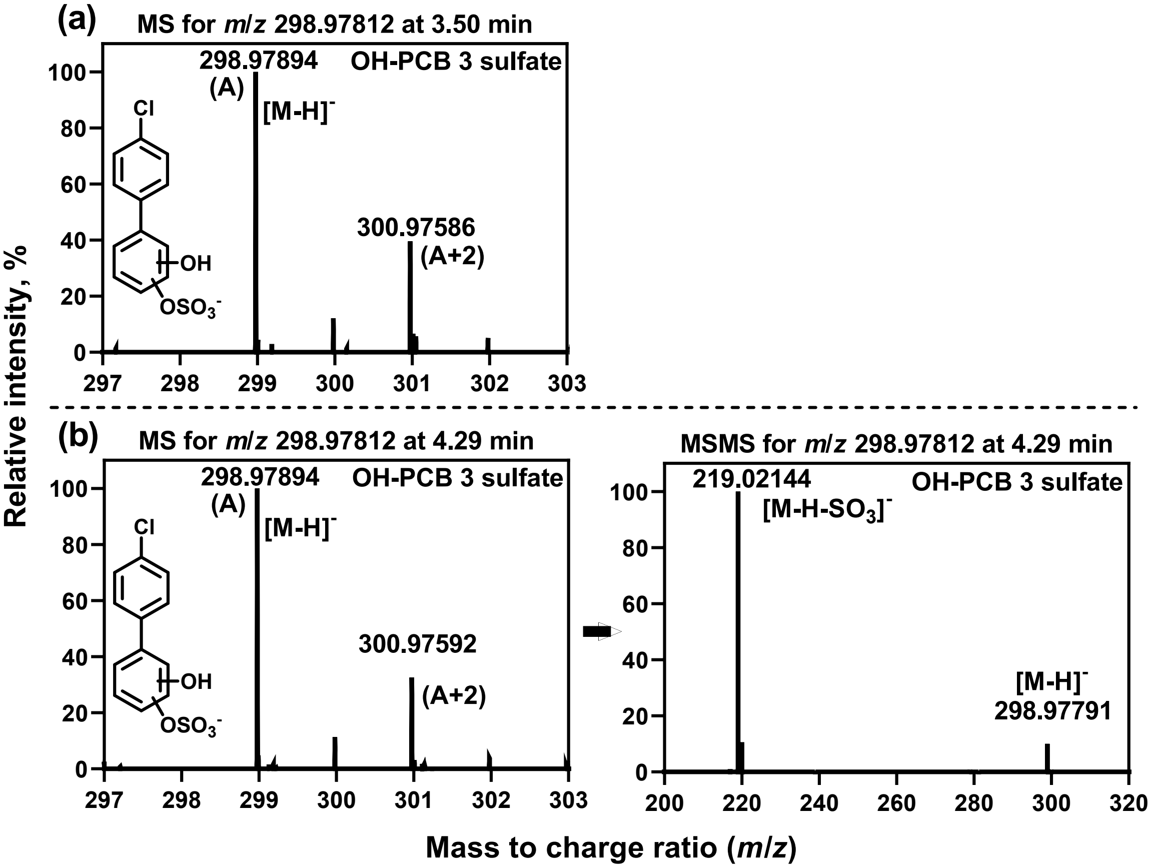

Fig. S6. LC-Orbitrap MS data with accurate masses of the molecular ion $[M-H]^-$, the isotope patterns consistent with a monochlorinated compound (A : A+2 = 1 : 3), and the MS/MS data support the formation of isomers of OH-PCB3 sulfate metabolites eluting at (a) 3.50 min and (b) 4.29 min in HepG2 cells exposed to PCB3. The MS/MS spectra showed characteristic fragment ions consistent with OH-PCB3 sulfates (i.e., loss of SO_3). The MS/MS data of the isomer eluting at 3.50 min was not collected due to its low concentration. The placement of the functional groups on phenyl rings is for illustration purposes only and does not indicate their actual position. The collision energies on the fragmentation cell for the MS/MS spectra was 30 eV; for additional details, see the text above.

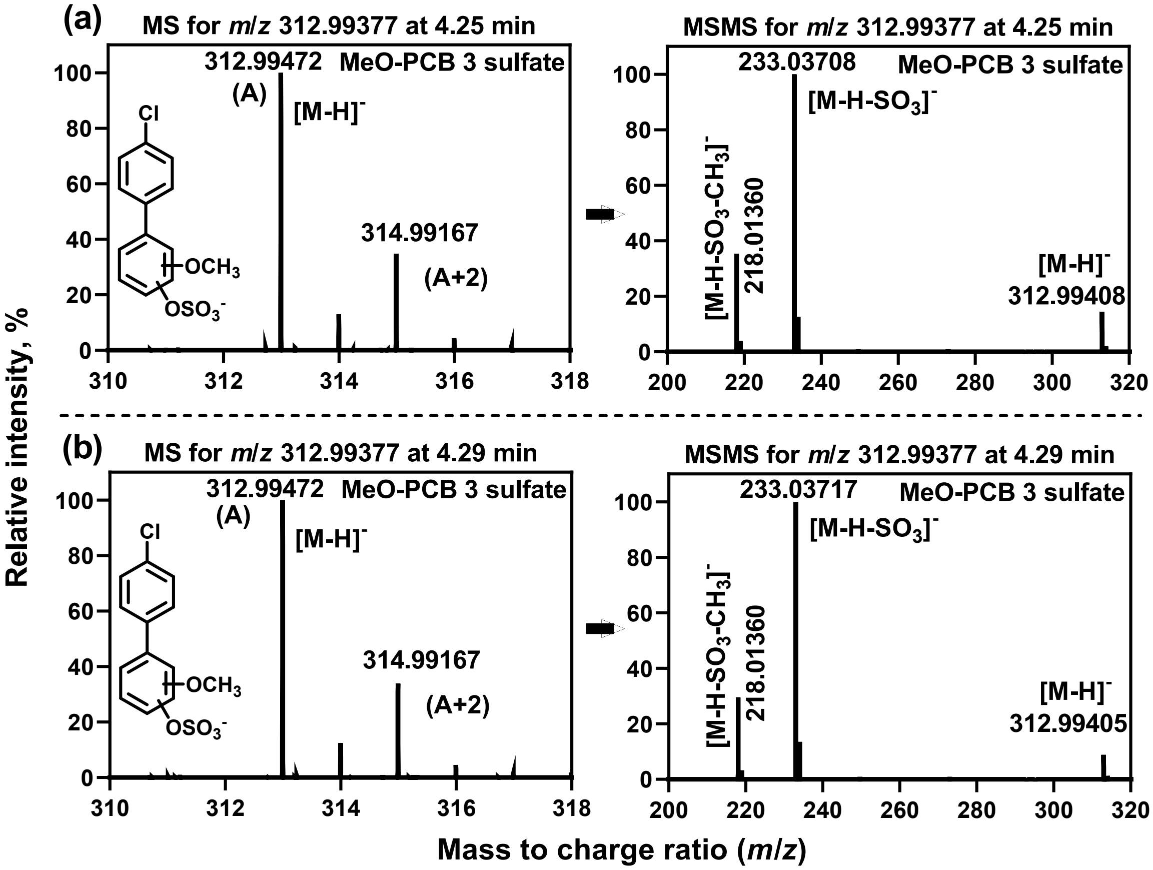

Fig. S7. LC-Orbitrap MS data with accurate masses of the molecular ion $[M-H]^-$, the isotope patterns consistent with a monochlorinated compound (A : A+2 = 1 : 3), and the MS/MS data support the formation of isomers of MeO-PCB3 sulfate metabolites eluting at (a) 4.25 min and (b) 4.29 min in HepG2 cells exposed to PCB3. The MS/MS spectra showed characteristic fragment ions consistent with MeO-PCB3 sulfates (i.e., loss of SO_3 and CH_3). The placement of the functional groups on phenyl rings is for illustration purposes only and does not indicate their actual position. The collision energies on the fragmentation cell for the MS/MS spectra was 30 eV; for additional details, see the text above.

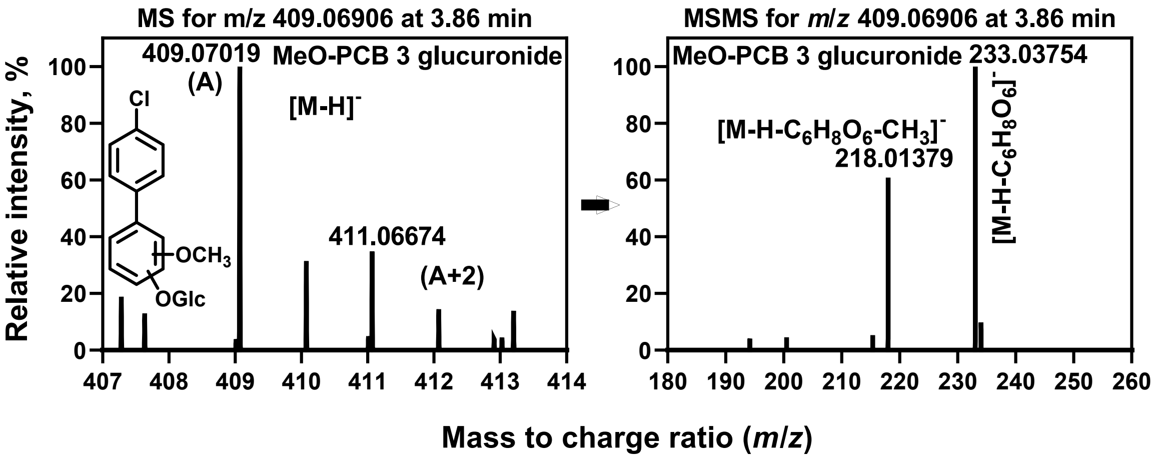

Fig. S8. LC-Orbitrap MS data with an accurate mass of the molecular ion $[M-H]^-$, the isotope pattern consistent with a monochlorinated compound (A : A+2 = 1 : 3), and the MS/MS data support the formation of an isomer of a MeO-PCB3 glucuronide metabolite eluting at 3.86 min in HepG2 cells exposed to PCB3. The MS/MS spectra showed featured characteristic fragment ions consistent with a MeO-PCB3 glucuronide (i.e., loss of $C_6H_8O_6$ and CH_3). The placement of the functional groups on phenyl rings is for illustration purposes only and does not indicate their actual position. The collision energies on the fragmentation cell for the MS/MS spectra was 30 eV; for additional details, see the text above.

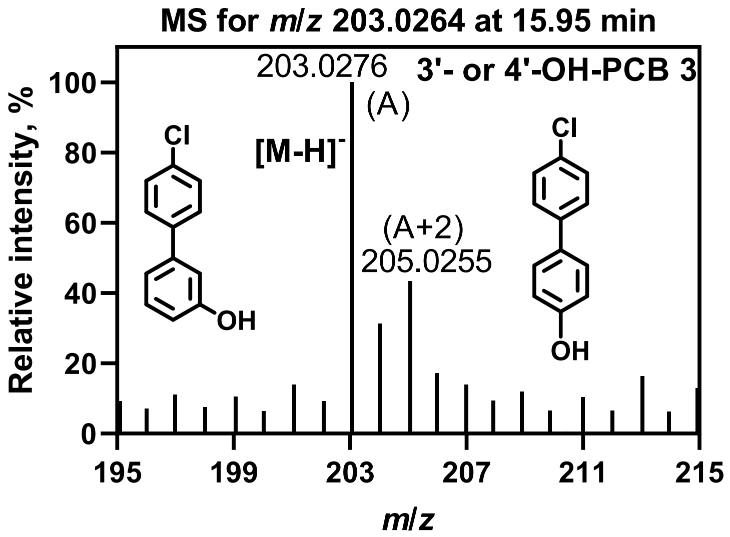

Fig. S9. LC-QTof MS data with an accurate mass of the molecular ion [M-H]$^-$ and the isotope pattern consistent with a monochlorinated compound (A : A+2 = 1 : 3) support the formation of a 3'- or 4'-OH-PCB3 metabolite by HepG2 cells exposed to PCB3. The retention time refers to the data listed in Table S4. For additional details, see the text above.

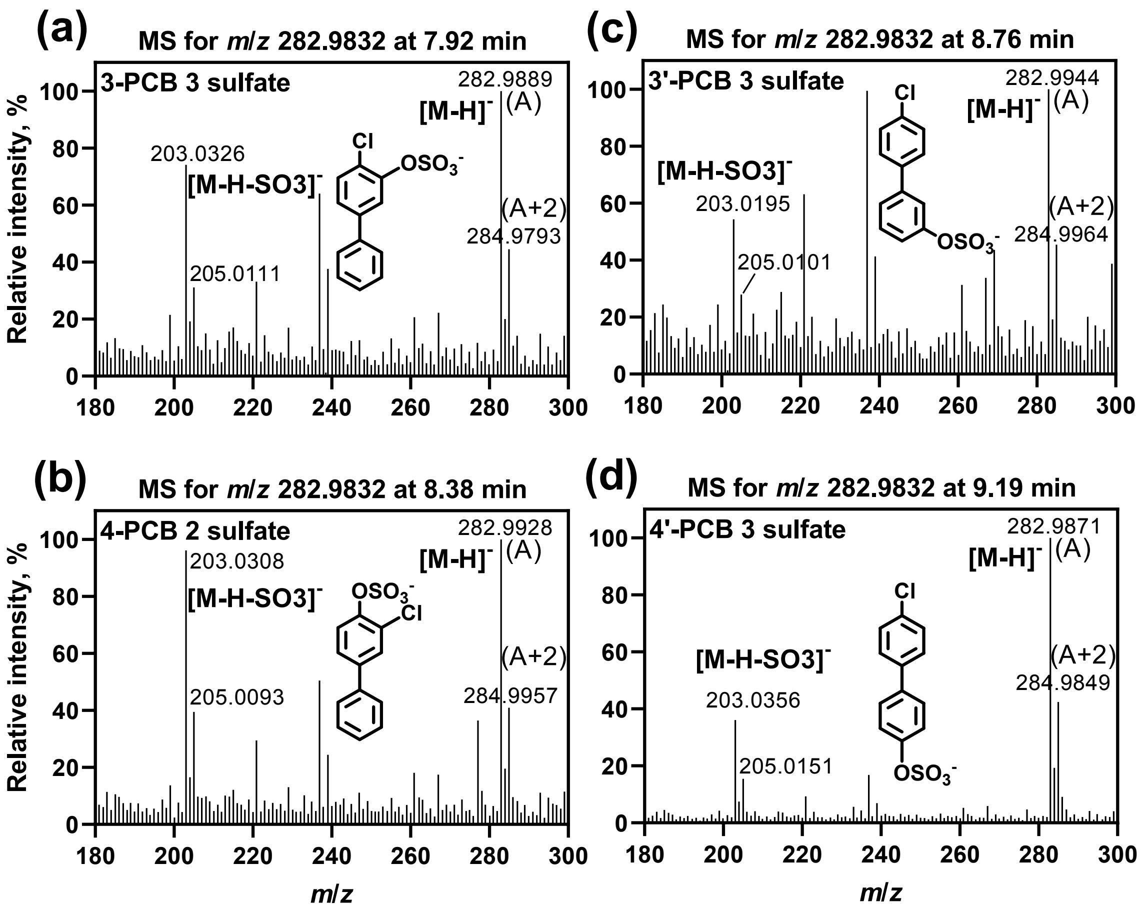

Fig. S10. LC-QTof MS data with accurate masses of the molecular ion [M-H]$^-$ and the isotope patterns consistent with a monochlorinated compound (A : A+2 = 1 : 3) support the formation of (a) 3-PCB3 sulfate, (b) 4-PCB2 sulfate, (c) 3'-PCB3 sulfate and (d) 4'-PCB3 sulfate by HepG2 cells exposed to PCB3. Fragment ions characteristic of PCB3 sulfate metabolites (i.e., loss of SO_3) were observed. The retention times refer to the data listed in Table S4. For additional details, see the text above.

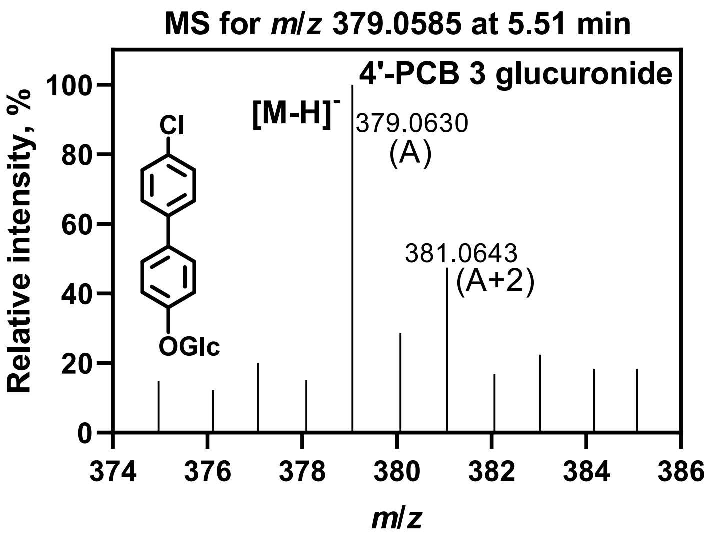

Fig. S11. LC-QTof MS data with an accurate mass of the molecular ion $[M-H]^-$ and the isotope pattern consistent with a monochlorinated compound (A : A+2 = 1 : 3) support the formation of a 4'-PCB3 glucuronide metabolite by HepG2 cells exposed to PCB3. The retention time refers to the data listed in Table S4. For additional details, see the text above.

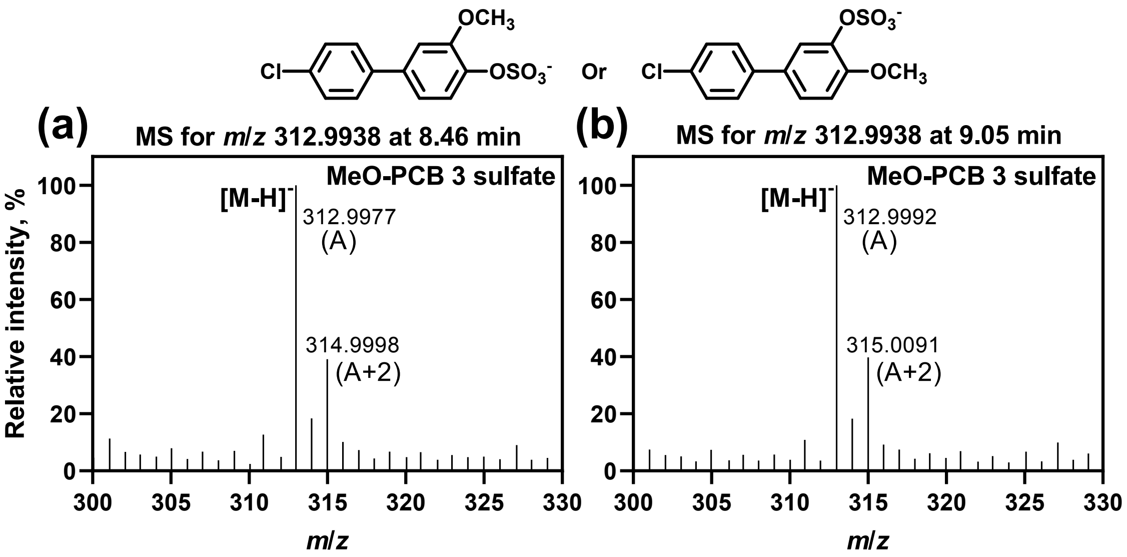

Fig. S12. LC-QTof data with accurate masses of the molecular ion [M-H]$^-$ and the isotope patterns consistent with a monochlorinated compound (A : A+2 = 1 : 3) support the formation of two isomers of MeO-PCB3 sulfate metabolites eluting at (a) 8.46 min and (b) 9.05 min in HepG2 cells exposed to PCB3. These two metabolites were identified as 3'-MeO-4'-PCB3 sulfate and 4'-MeO-3'-PCB3 sulfate. The retention times refer to the data listed in Table S4. For additional details, see the text above.

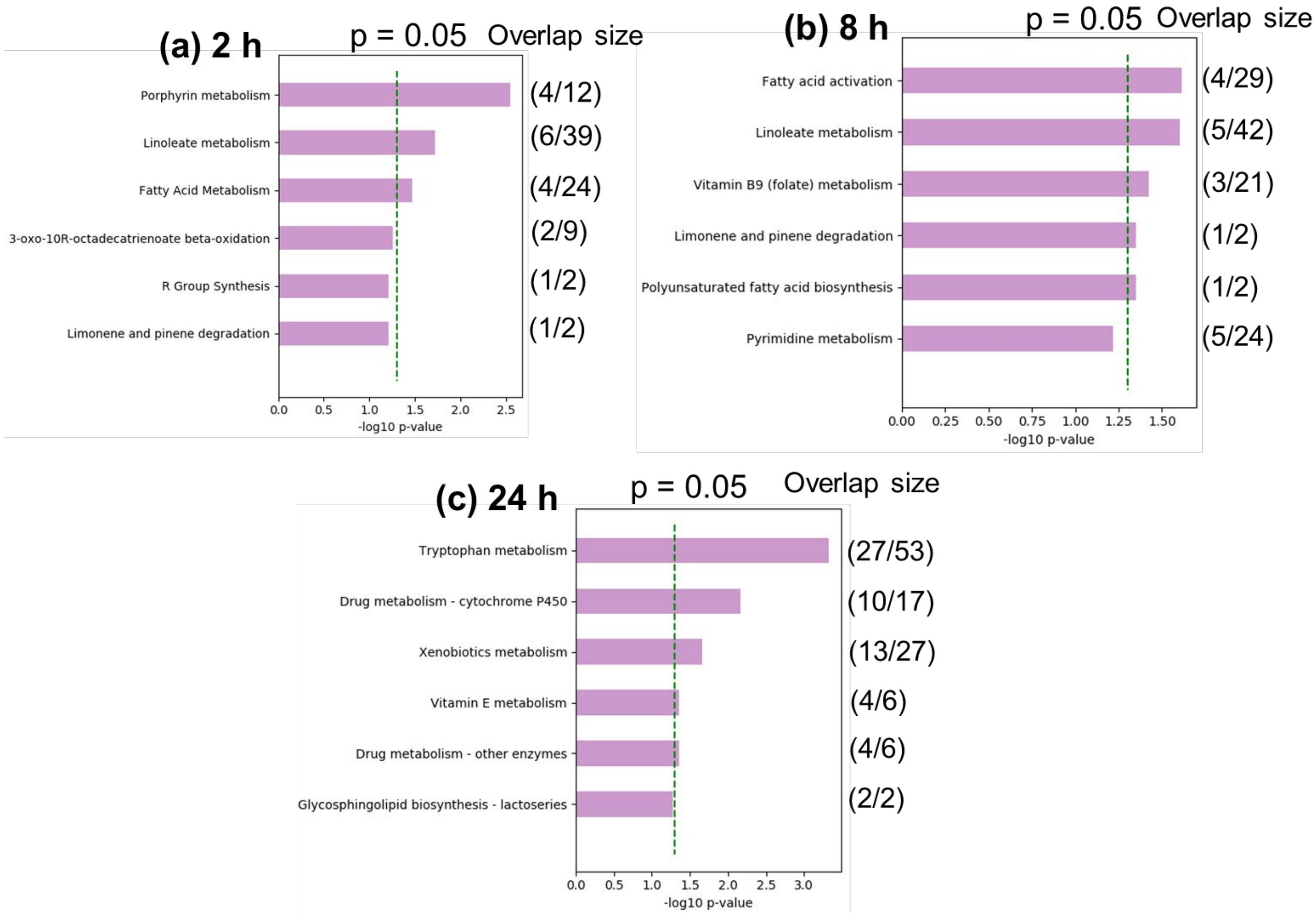

Fig. S13. Pathway enrichment analysis revealed several endogenous metabolic pathways were altered in HepG2 cells following PCB 3 exposure for (a) 2 h, (b) 8 h, and (c) 24 h. Analyses were performed with mummichog with parameter setting allowing features to be included as significant if their primary molecular ion $[M-H]^-$ or one of the molecular adduct ions ($[M\ (13C)-H]^-$, $[M+Na-2H]^-$, $[M+ACN-H]^-$ and $[M-H2O-H]^-$) statistically differs between exposed and control groups. For pathway enrichment analysis with more stringent parameter settings, see Fig. 4.

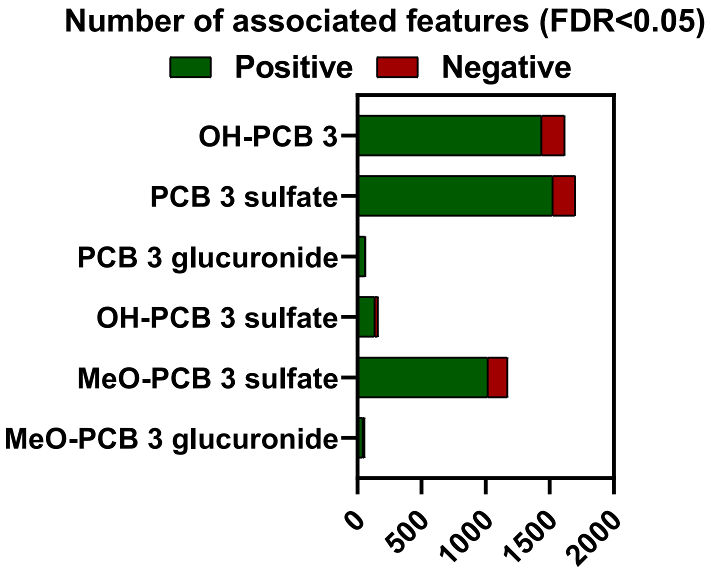

Fig. S14. A metabolome-wide association study with six PCB3 metabolite classes suggests that OH-PCB3, PCB3 sulfate, and MeO-PCB3 sulfate have broader effects on the HepG2 cell metabolome than other metabolite classes. Analyses were performed on 18 samples incubated with and without PCB3. The peak areas of the PCB3 metabolites were normalized by the total intensities and log_2 transformed. The association analyses between each PCB3 metabolite class and the metabolome were conducted using a linear model. Significantly associated metabolites were selected with a false discovery rate (FDR) below 0.05. For more details about the data analyses, see the text above.

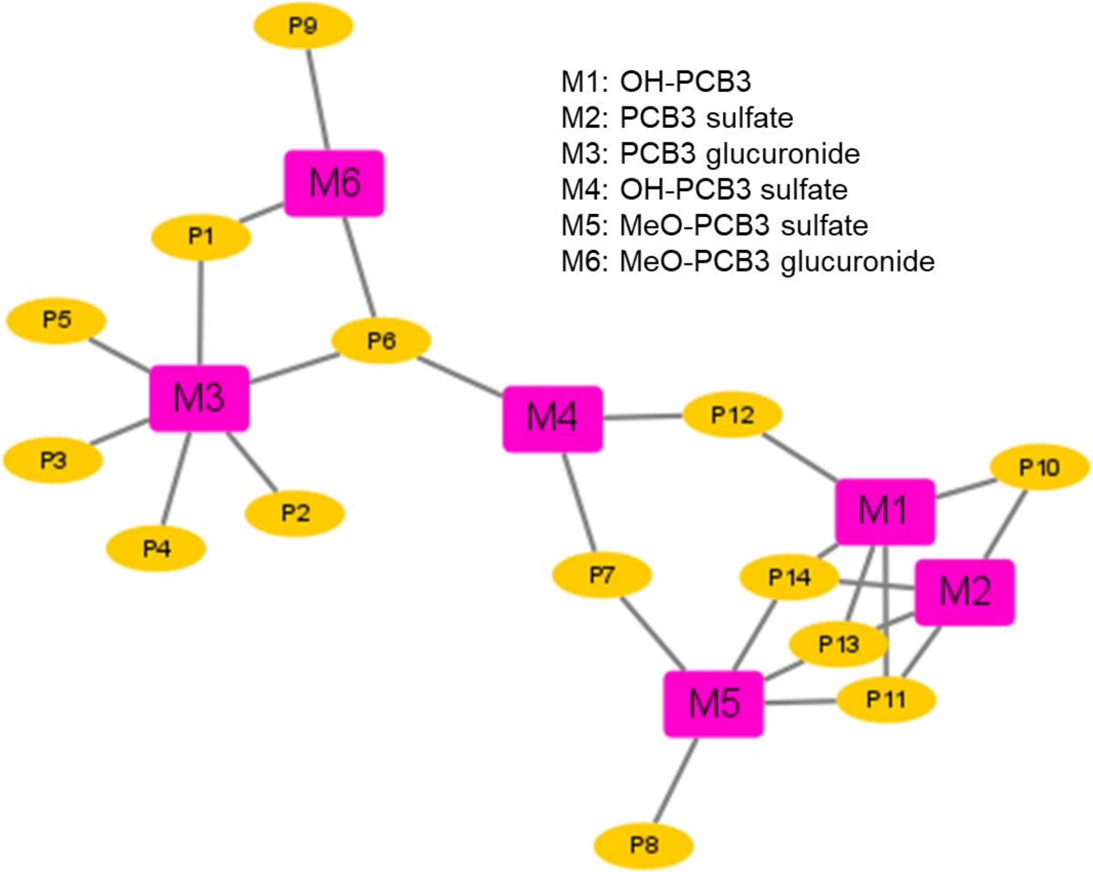

Fig. S15. Network correlation analysis revealed co-effects of PCB3 metabolite classes on the endogenous metabolic pathways. Metabolic pathways were shown only when they were associated with at least one PCB3 metabolite class with p-values below 0.05. P1: Bile acid biosynthesis; P2: Tyrosine metabolism; P3: Alkaloid biosynthesis II; P4: TCA cycle; P5: Vitamin B6 (pyridoxine) metabolism; P6: Valine, leucine and isoleucine degradation; P7: Phytanic acid peroxisomal oxidation; P8: Drug metabolism - cytochrome P450; P9: Vitamin D3 (cholecalciferol) metabolism; P10: Proteoglycan biosynthesis; P11: 3-oxo-10R-octadecatrienoate beta-oxidation; P12: Tryptophan metabolism; P13: Vitamin A (retinol) metabolism; P14: Lysine metabolism.

References

1. McLean, M.R.; Bauer, U.; Amaro, A.R.; Robertson, L.W. Identification of catechol and hydroquinone metabolites of 4-monochlorobiphenyl. *Chem. Res. Toxicol.* **1996,** *9*, 158-164.

2. Li, X.S.; Parkin, S.; Duffel, M.W.; Robertson, L.W.; Lehmler, H.J. An efficient approach to sulfate metabolites of polychlorinated biphenyls. *Environ. Int.* **2010,** *36*, 843-848.

3. Zhai, G.S.; Lehmler, H.J.; Schnoor, J.L. New hydroxylated metabolites of 4-monochlorobiphenyl in whole poplar plants. *Chem. Cent. J.* **2011,** *5*.

4. Dhakal, K.; He, X.R.; Lehmler, H.J.; Teesch, L.M.; Duffel, M.W.; Robertson, L.W. Identification of sulfated metabolites of 4-chlorobiphenyl (PCB3) in the serum and urine of male rats. *Chem. Res. Toxicol.* **2012,** *25*, 2796-2804.

5. Zhang, C.-Y.; Flor, S.; Ruiz, P.; Dhakal, R.; Hu, X.; Teesch, L.M.; Ludewig, G.; Lehmler, H.-J. 3,3'-Dichlorobiphenyl is metabolized to a complex mixture of oxidative metabolites, including novel methoxylated metabolites, by HepG2 cells. *Environ. Sci. Technol.* **2020,** *54*, 12345-12357.

6. Uwimana, E.; Ruiz, P.; Li, X.S.; Lehmler, H.J. Human CYP2A6, CYP2B6, AND CYP2E1 atropselectively metabolize polychlorinated biphenyls to hydroxylated metabolites. *Environ. Sci. Technol.* **2019,** *53*, 2114-2123.

7. Kaminsky, L.S.; Kennedy, M.W.; Adams, S.M.; Guengerich, F.P. Metabolism of dichlorobiphenyls by highly purified isozymes of rat-liver cytochrome-P-450. *Biochemistry* **1981,** *30*, 577-588.

8. Warner, N.A.; Martin, J.W.; Wong, C.S. Chiral polychlorinated biphenyls are biotransformed enantioselectively by mammalian cytochrome P-450 isozymes to form hydroxylated metabolites. *Environ. Sci. Technol.* **2009,** *43*, 114-121.

9. Song, Y.; Wagner, B.A.; Lehmler, H.J.; Buettner, G.R. Semiquinone radicals from oxygenated polychlorinated biphenyls: Electron paramagnetic resonance studies. *Chem. Res. Toxicol.* **2008,** *21*, 1359-1367.

10. Amaro, A.R.; Oakley, G.G.; Bauer, U.; Spielmann, H.P.; Robertson, L.W. Metabolic activation of PCBs to quinones: Reactivity toward nitrogen and sulfur nucleophiles and influence of superoxide dismutase. *Chem. Res. Toxicol.* **1996,** *9*, 623-629.

11. Li, X.; Liu, Y.; Martin, J.W.; Cui, J.Y.; Lehmler, H.J. Nontarget analysis reveals gut microbiome-dependent differences in the fecal PCB metabolite profiles of germ-free and conventional mice. *Environ. Pollut.* **2020,** *268*, 115726.

12. Zhang, C.-Y.; Flor, S.; Ludewig, G.; Lehmler, H.-J. Atropselective partitioning of polychlorinated biphenyls in a HepG2 cell culture system: experimental and modeling results. *Environ. Sci. Technol.* **2020,** *54*, 13817-13827.

13. Hu, X.; Lehmer, H.J.; Adamcakova-Dodd, A.; Thorne, P.S. Elimination of inhaled 3,3 ‘-dichlorobiphenyl and the formation of the 4-hydroxylated metabolite. *Environ. Sci. Technol.* **2013,** *47*, 4743-4751.

14. Yu, T.W.; Park, Y.; Johnson, J.M.; Jones, D.P. apLCMS-adaptive processing of high-resolution LC/MS data. *Bioinformatics* **2009,** *25*, 1930-1936.

15. Uppal, K.; Soltow, Q.A.; Strobel, F.H.; Pittard, W.S.; Gernert, K.M.; Yu, T.W.; Jones, D.P. xMSanalyzer: automated pipeline for improved feature detection and downstream

analysis of large-scale, non-targeted metabolomics data. *BMC Bioinformatics* **2013,** *14*, 15.

16. Go, Y.M.; Walker, D.I.; Soltow, Q.A.; Uppal, K.; Wachtman, L.M.; Strobel, F.H.; Pennell, K.; Promislow, D.E.L.; Jones, D.P. Metabolome-wide association study of phenylalanine in plasma of common marmosets. *Amino Acids* **2015,** *47*, 589-601.
17. Benjamini, Y.; Hochberg, Y. Controlling the false discovery rate - a practical and powerful approach to multiple testing. *J. R. Stat. Soc. B* **1995,** *57*, 289-300.
18. Li, S.Z.; Park, Y.; Duraisingham, S.; Strobel, F.H.; Khan, N.; Soltow, Q.A.; Jones, D.P.; Pulendran, B. Predicting network activity from high throughput metabolomics. *Plos Comput. Biol.* **2013,** *9*, e1003123.
19. Uppal, K.; Walker, D.I.; Jones, D.P. xMSannotator: An R package for network-based annotation of high-resolution metabolomics data. *Anal. Chem.* **2017,** *89*, 1063-1067.
20. Wishart, D.S.; Jewison, T.; Guo, A.C.; Wilson, M.; Knox, C.; Liu, Y.F.; Djoumbou, Y.; Mandal, R.; Aziat, F.; Dong, E.; Bouatra, S.; Sinelnikov, I.; Arndt, D.; Xia, J.G.; Liu, P.; Yallou, F.; Bjorndahl, T.; Perez-Pineiro, R.; Eisner, R.; Allen, F.; Neveu, V.; Greiner, R.; Scalbert, A. HMDB 3.0-The human metabolome database in 2013. *Nucleic Acids Res.* **2013,** *41*, D801-D807.
21. Kanehisa, M.; Furumichi, M.; Tanabe, M.; Sato, Y.; Morishima, K. KEGG: new perspectives on genomes, pathways, diseases and drugs. *Nucleic Acids Res.* **2017,** *45*, D353-D361.
22. Shen, X.T.; Wang, R.H.; Xiong, X.; Yin, Y.D.; Cai, Y.P.; Ma, Z.J.; Liu, N.; Zhu, Z.J. Metabolic reaction network-based recursive metabolite annotation for untargeted metabolomics. *Nat. Commun.* **2019,** *10*.

23. Horai, H.; Arita, M.; Kanaya, S.; Nihei, Y.; Ikeda, T.; Suwa, K.; Ojima, Y.; Tanaka, K.; Tanaka, S.; Aoshima, K.; Oda, Y.; Kakazu, Y.; Kusano, M.; Tohge, T.; Matsuda, F.; Sawada, Y.; Hirai, M.Y.; Nakanishi, H.; Ikeda, K.; Akimoto, N.; Maoka, T.; Takahashi, H.; Ara, T.; Sakurai, N.; Suzuki, H.; Shibata, D.; Neumann, S.; Iida, T.; Tanaka, K.; Funatsu, K.; Matsuura, F.; Soga, T.; Taguchi, R.; Saito, K.; Nishioka, T. MassBank: a public repository for sharing mass spectral data for life sciences. *J. Mass. Spectrom.* **2010,** *45*, 703-714.

24. Schymanski, E.L.; Jeon, J.; Gulde, R.; Fenner, K.; Ruff, M.; Singer, H.P.; Hollender, J. Identifying small molecules via high resolution mass spectrometry: communicating confidence. *Environ. Sci. Technol.* **2014,** *48*, 2097-2098.

25. Marek, R.F.; Thome, P.S.; Herkert, N.J.; Awad, A.M.; Hornbuckle, K.C. Airborne PCBs and OH-PCBs inside and outside urban and rural US schools. *Environ. Sci. Technol.* **2017,** *51*, 7853-7860.

26. Persoon, C.; Peters, T.M.; Kumar, N.; Hornbuckle, K.C. Spatial distribution of airborne polychlorinated biphenyls in Cleveland, Ohio and Chicago, Illinois. *Environ. Sci. Technol.* **2010,** *44*, 2797-2802.
